# *Mtb* HLA-E-tetramer-sorted CD8^+^ T cells have a diverse TCR repertoire

**DOI:** 10.1016/j.isci.2024.109233

**Published:** 2024-02-15

**Authors:** Linda Voogd, Anne M.H.F. Drittij, Calinda K.E. Dingenouts, Kees L.M.C. Franken, Vincent van Unen, Krista E. van Meijgaarden, Paula Ruibal, Renate S. Hagedoorn, Judith A. Leitner, Peter Steinberger, Mirjam H.M. Heemskerk, Mark M. Davis, Thomas J. Scriba, Tom H.M. Ottenhoff, Simone A. Joosten

**Affiliations:** 1 Department of Infectious Diseases, Leiden University Medical Center, Leiden, the Netherlands; 2 Institute of Immunity, Transplantation and Infection, Stanford University School of Medicine, Palo Alto, CA, USA; 3 Department of Hematology, Leiden University Medical Center, Leiden, the Netherlands; 4 Centre for Pathophysiology, Infectiology and Immunology, Institute of Immunology, Medical University of Vienna, Vienna, Austria; 5 Howard Hughes Medical Institute, Stanford University School of Medicine, Palo Alto, CA, USA; 6 South African Tuberculosis Vaccine Initiative, Institute of Infectious Disease and Molecular Medicine and Division of Immunology, Department of Pathology, University of Cape Town, Cape Town, South Africa

**Keywords:** Genotyping, Immunology, Transcriptomics

## Abstract

HLA-E molecules can present self- and pathogen-derived peptides to both natural killer (NK) cells and T cells. T cells that recognize HLA-E peptides via their T cell receptor (TCR) are termed donor-unrestricted T cells due to restricted allelic variation of HLA-E. The composition and repertoire of HLA-E TCRs is not known so far. We performed TCR sequencing on CD8^+^ T cells from 21 individuals recognizing HLA-E tetramers (TMs) folded with two *Mtb*-HLA-E-restricted peptides. We sorted HLA-E *Mtb* TM^+^ and TM^−^ CD8^+^ T cells directly *ex vivo* and performed bulk RNA-sequencing and single-cell TCR sequencing. The identified TCR repertoire was diverse and showed no conservation between and within individuals. TCRs selected from our single-cell TCR sequencing data could be activated upon HLA-E/peptide stimulation, although not robust, reflecting potentially weak interactions between HLA-E peptide complexes and TCRs. Thus, HLA-E-*Mtb*-specific T cells have a highly diverse TCR repertoire.

## Highlights

• CD8^+^ TCRs recognizing HLA-E/*Mtb* tetramers are diverse within and between individuals
• Both Vα and Vβ chains of HLA-E TCRs recognize the HLA-E/*Mtb* complex
• CD8^+^ TCRs recognizing HLA-E/*Mtb* tetramers have limited clonal expansions

## Graphical abstract

**Figure:**
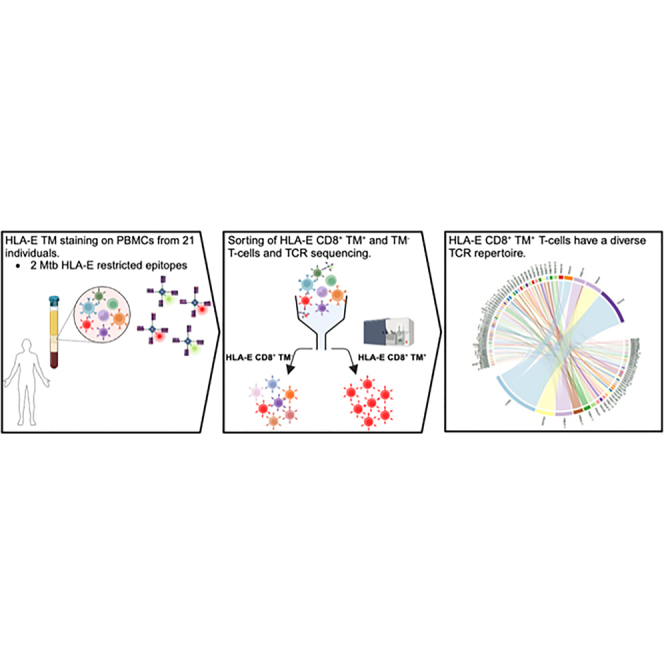

## Introduction

Despite large-scale vaccination efforts with the Bacillus Calmette-Guerin (BCG) vaccine, infection with *Mycobacterium tuberculosis* (*Mtb*) and the subsequent development of tuberculosis disease (TB) remains a global burden.^1^ The search for and development of improved vaccines is therefore urgently needed.^2^ Initiation of CD4^+^ and CD8^+^ T cell responses via presentation of peptides by HLA-II and HLA-I molecules, respectively, is an essential component in controlling *Mtb* infection and preventing progression to active TB.^3^ A major challenge in peptide-based T-cell-targeting vaccine development is the large interindividual allelic heterogeneity in classical HLA alleles, which complicates the search for *Mtb* peptides that can induce T cell responses within a genetically diverse population. In contrast, a subclass of HLA-I molecules, called non-classical HLA-I (i.e., HLA-Ib), have limited allelic variations and include HLA-E, -F, and -G. Although several alleles have been identified for HLA-F and -G, HLA-E has only two functional alleles, HLA-E^∗^01:01 and ^∗^01:03, that differ in one amino acid located outside the peptide-binding groove (PBG). This very limited allelic variation justifies the classification of HLA-E as a monomorphic molecule.^4^^,^^5^^,^^6^^,^^7^ HLA-E is present at different intracellular compartments and is more unstable at the cell surface compared with classical HLA-I molecules.^8^

HLA-E was first discovered as a ligand for the CD94/NKG2A(C) co-receptor expressed on natural killer (NK) cells to regulate NK-cell-mediated lysis.^9^^,^^10^^,^^11^ Later, it was discovered that HLA-E can accommodate a repertoire of sequentially and conformationally divergent peptides in the PBG and that HLA-E can present these peptides to T cell receptors (TCRs) in *Mtb*, human immunodeficiency virus (HIV), *Cytomegalovirus* (CMV), and *Salmonella* typhimurium infections, as well as several human tumors.^12^^,^^13^^,^^14^^,^^15^^,^^16^^,^^17^^,^^18^ CD8^+^ T cell clones specific for two *Mtb*-derived HLA-E-restricted peptides were generated using limiting dilution cultures. These T cell clones could be activated following *Mtb* peptide stimulation as measured by CD137 upregulation and Zap70 activation, showing antigen-specific TCR activation. Moreover, most of these HLA-E-*Mtb*-restricted T cell clones, although to varying degrees, could lyse BCG-infected monocytes and control intracellular mycobacterial outgrowth in *Mtb*-infected macrophages.^12^ Besides the cytolytic and suppressive functions, the phenotype of HLA-E-*Mtb*-specific T cells revealed to be unorthodox relative to classical HLA-I T cells as they could secrete both T helper 1- and 2-associated cytokines.^12^^,^^13^ These findings indicate that HLA-E restricted *Mtb*-specific T cells can contribute to functional effector responses.

HLA-E is further conserved among vertebrate species with orthologues found in mice (Qa-1^b^), rhesus macaques (RMs) (Mamu-E), and cynomolgus macaques (Mafa-E), facilitating rapid translation of pre-clinal research to humans.^19^ The potential of HLA-E as a vaccination target *in vivo* was demonstrated in RMs against the *simian immunodeficiency virus* (SIV) and in mice against *Mtb* infection and development of TB disease.^20^^,^^21^^,^^22^^,^^23^ Vaccination with an SIV/rhesus CMV strain 68-1 vector expressing the SIVgag protein resulted in broad and effective Mamu-E CD8^+^ T cell responses in nearly all vaccinated RMs, and Qa-1^b^ T cell responses in *Mtb*-infected mice were essential to control and protect against *Mtb*. Altogether, these studies suggest that HLA-E is a promising vaccination target for *Mtb* and other pathogens to induce protection across a genetically diverse population.

Recently, the improvement of predictive HLA-E peptide-binding algorithms enabled the identification of high-affinity *Mtb* peptides.^17^ Next to peptide identification, we investigated the HLA-E-*Mtb*-restricted T cell repertoire in more detail to deepen our current understanding on the diversity of HLA-E T cells in general and in TB specifically*.* T cells that recognize monomorphic molecules are called donor unrestricted T cells (DURTs) and generally express invariant TCRs that are possibly conserved across individuals.^24^^,^^25^ Previous studies on cytolytic T cell clones recognizing conserved HLA-E-restricted UL40-derived epitopes from CMV in four donors revealed a nearly monoclonal expansion in the TRBV region, which was different between donors.^15^^,^^26^ However, more recently, TCR profiling on 13 HLA-E-restricted T cell clones from one donor recognizing the HLA-E-restricted HIV-derived peptide RL9HIV and on 13 HLA-E-restricted T cell clones from two donors specific for Sars-CoV-2-derived HLA-E peptide binders revealed a more diverse TCR repertoire with variations between clones in the usage and pairing of variable (V) and joining (J) fragments in the TCRα and β chains.^27^^,^^28^ Thus, the question remains if HLA-E-restricted TCRs can be considered invariant, like other DURTs, or are diverse.

In this study, we performed TCR repertoire as well as RNA-sequencing analysis on CD8^+^ T cells sorted with HLA-E TMs folded with two *Mtb*-derived HLA-E-specific peptides in 21 South African individuals.^16^ We show that the TCR repertoire of *Mtb* HLA-E TM^+^ T cells is diverse across and within individuals, with no indications for conservation of TCRs between individuals. We further show that HLA-E *Mtb* TM^+^ TCRs selected using GLIPH2 could be activated after stimulation with the HLA-E *Mtb* peptides in both Jurkat cells and primary T cells using Zap70 phosphorylation as a readout, although with relatively low signals compared with classical HLA-I-restricted T cells.

## Results

### HLA-E *Mtb* TM^+^ CD8^+^-sorted TCRs contain heterogeneous variable and joining α and β fragments

To unravel the TCR repertoire of HLA-E-restricted *Mtb*-specific CD8^+^ T cells, we stained PBMCs from 21 South African individuals with HLA-E^∗^01:03 tetramers (TMs) folded with two *Mtb*-derived peptides that are established binders to HLA-E^∗^01:03, called p34 and p55. The gating strategies for sorting 1,000–2,000 HLA-E TM^+^ CD8^+^ T cells are shown in Figure S1A. We validated correct folding of the HLA-E^∗^01:03 monomers with HPLC and tetramerization and integrity of the monomers using LILRB1 staining.^18^^,^^29^ In our staining and sorting approach, we always combined two unrelated HLA-E TMs (e.g., p34 and p55 or p34 and another published *Mtb*-specific HLA-E-restricted peptide^16^) with different fluorochromes in the same staining to control for possible a-specific staining of the HLA-E TMs. Double-positive cells were considered a-specific and only single-positive cells were sorted (Figures S1B and S1C; Table S1 for TM/peptide combinations). HLA-E TM^+^ cells were either sorted in bulk or as single cells and were used for downstream RNA-seq (bulk) and TCR-seq (single cells) analysis. We initially wanted to compare the sequencing data of p34 and p55 HLA-E TM^+^ T cells with the well described and characterized HLA-E-restricted UL40-derived epitope from CMV; however, only two individuals in our cohort showed detectable HLA-E UL40 TM staining, which was the reason we compared the sorted p34 and p55 HLA-E TM^+^ T cells with the total CD8^+^ T cell pool (i.e., CD8^+^ T cells not sorted based on HLA-E TM staining for these two *Mtb* epitopes, called CD8^+^ TM^−^ T cells).

**Figure 1:**
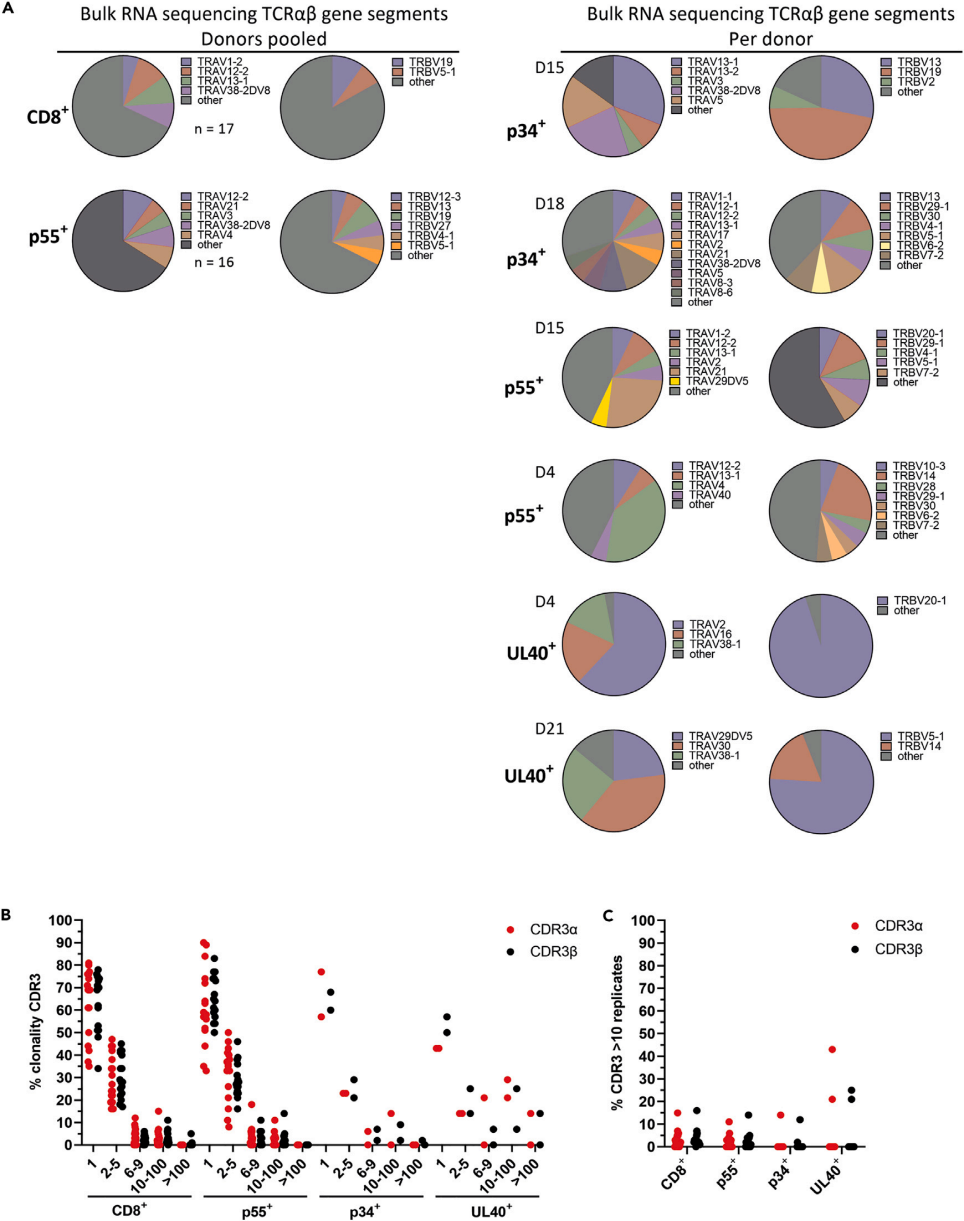
Sorted *Mtb* HLA-E TM^+^ CD8^+^ T cells have a diverse TCR repertoire TCR encoding RNA transcripts were extracted from the total RNA sequencing dataset by *in silico* alignment using MiXCR. (A) Pie charts show the frequency of TRAV and TRBV variable fragment usage in both pooled donors (left) and per donor (right). Fragments present in ≥5% of all TCRα/β in pooled donors (CD8^+^ TM^−^ n = 17, p55^+^ n = 16) or ≥5% of all TCRα/β per donor (p34^+^ n = 2, UL40^+^ n = 2, p55^+^ n = 2) are named, and the remaining fragments are summarized as “other”. (B) Frequency of CDR3αβ sequences present as a single copy or with 2–5, 6–9, 10–100, or >100 copies. Each dot represents the frequency of CDR3 sequences in a donor with that clonal number. Data are depicted from n = 17 total CD8^+^ TM^−^ samples, n = 16 TMp55^+^-sorted samples, n = 2 TMp34^+^, and n = 2 TMUL40^+^-sorted samples. (C) The percentage of CDR3αβ sequences present with ≥10 copies per donor per population as stated on the y axis.

TCRαβ fragments were extracted from bulk RNA-seq data and from single-cell sequencing data with the algorithm MiXCR. MiXCR identifies single Vα, Vβ, Jα, Jβ, and CDR3αβ fragments from sequence data via sequence alignments. Bulk RNA-seq on CD8^+^ TM^−^ T cells from 17 donors combined revealed a heterogeneous TCR repertoire, as expected (Figure 1A, left; Table S2). Both Vα, Vβ and Jα, Jβ fragments were diverse in these CD8^+^ TM^−^ T cells as reflected by the low frequencies of single Vα and Vβ fragments (data not shown). Bulk RNA-seq on p55 HLA-E TM^+^-sorted T cells from 16 donors combined revealed a similarly diverse Vα and Vβ repertoire (Figure 1A, left). Likewise, bulk RNA-seq analysis per donor on p34 and p55 HLA-E TM^+^-sorted T cells revealed a diverse TCR repertoire as well albeit with a certain degree of preference per donor for certain individual Vα or Vβ fragments, as shown for the two representative donors in Figure 1A, right panel. Although only two donors had sufficient UL40 HLA-E TM^+^-sorted T cells, the Vα and Vβ fragments were more homogeneous/conserved, particularly in the Vβ fragments (Figure 1A, right). In addition, there was a different Vβ fragment preferentially used between the two donors: TRBV20-1 in donor 4 and TRBV5-1 and -14 in donor 21.

In addition to Vαβ fragment identification from the bulk RNA-seq data, we also assessed the conservation of the CDR3α and β region via clonotype identification using MiXCR on the bulk RNA-seq data (Figure 1B). The majority of CDR3α and β sequences in the CD8^+^ TM^−^ T cell population (17 donors), including the p34 (2 donors) and p55 (16 donors) HLA-E TM^+^ T cell populations, were present with a copy number of 1–5, suggesting that the clonality of the CDR3 sequences was low, indicating diversity in these populations (Figure 1B). In addition, the frequency of possibly clonally expanded (copy number of 10 or more) CDR3α and β sequences was low as well in these populations for each donor (Figure 1C). However, compared with p34 and p55 HLA-E TM^+^-sorted TCRs, UL40 HLA-E TM^+^-sorted TCRs had a higher frequency of CDR3αβ sequences with a copy number of 10 or more, suggesting a more clonally expanded T cell population (Figure 1C). As such, the heterogeneity in the CDR3αβ and Vα, β regions of *Mtb* HLA-E TM^+^ TCRs, but not in UL40 HLA-E TM^+^ TCRs, suggests that HLA-E *Mtb* TM^+^ TCRs are more diverse as determined from the RNA-seq data.

**Figure 2:**
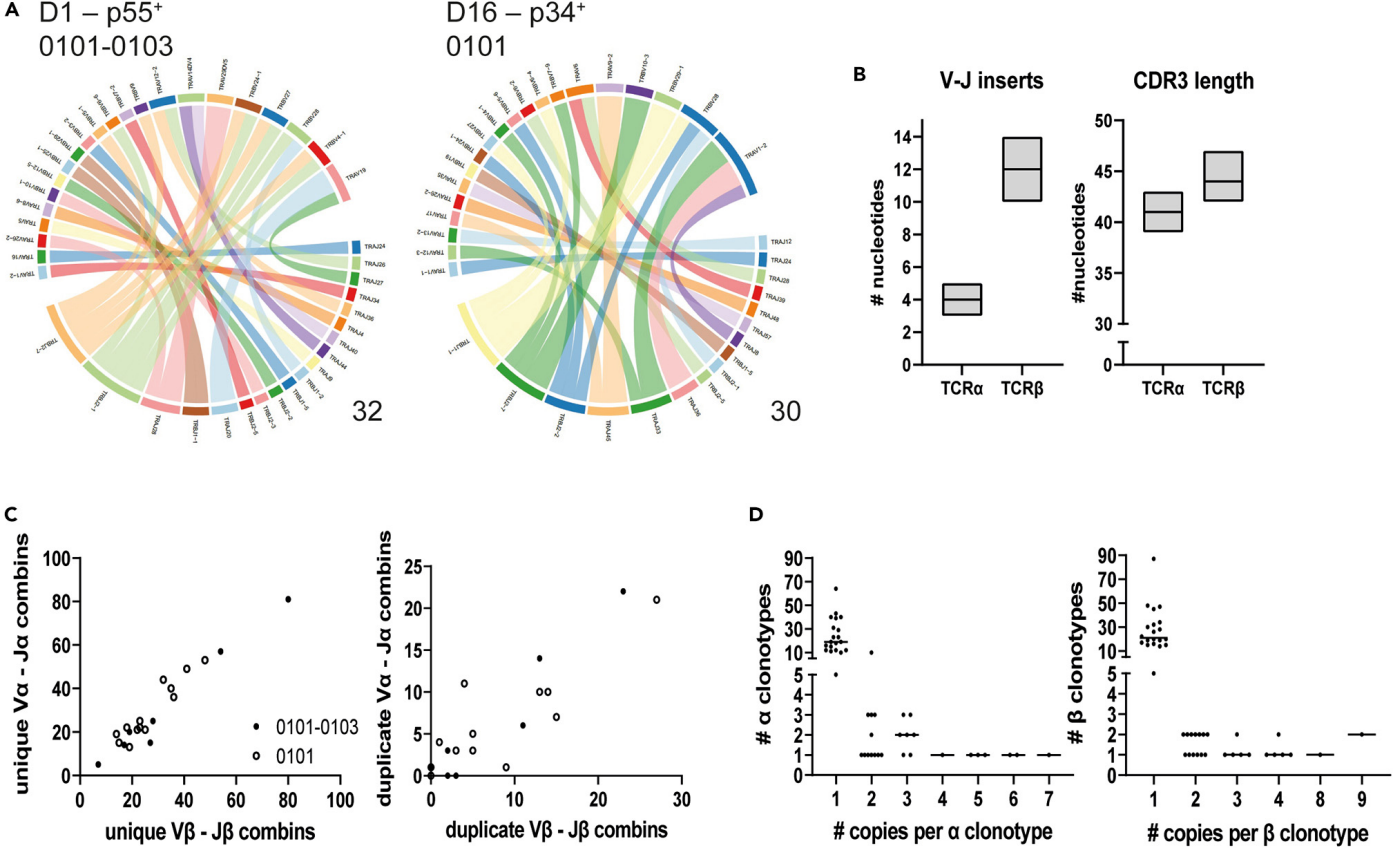
VDJ pairs in sorted p34 and p55 HLA-E TM^+^ T cells have a diverse character (A) Donor-specific variation in V-J segment pairing in CDR3 junctions for TCRα and β chain in *Mtb* TM^+^ CD8^+^ T cell populations. A single TCRα and β chain VDJ recombination was performed on single-cell-sorted HLA-E p34 and p55 TM^+^ T cells. Representative plots for p55^+^ (left) and p34^+^ (right) populations in respectively donor 1 (^∗^01:01–^∗^01:03) and donor 16 (^∗^01:01) are shown. The chord diagram connects segment pairs by corresponding V-J pair frequency. The total number of identified CDR3s is respectively 32 and 30. (B) CDR3 and insert length in number of nucleotides. Variation in length and corresponding median length of the CDR3 nucleotide sequence (left) and the median number of inserted random nucleotides in the CDR3 sequence (right) were calculated using the BasicStats function of VDJ tools R plug (n = 17). Box shows the median lengths/inserts min to max. (C) Variation in TCRα and β chain concerning V-J segment pairing. Number of unique V-J pairings (left) or duplicate pairings (right) was scored for TM^+^ populations per donor (n = 17). The total number of CDR3s was dependent on sequence sample size. Heterozygous HLA-E^∗^01:01-^∗^01:03 donors are indicated by filled dots; homozygous HLA-E^∗^01:01 donors are open (no HLA-E^∗^01:03 donors identified). (D) Distribution of repetitive α (left) and β (right) clonotypes over different donors. Frequency of CDR3 clonotype replicates on amino acid sequence divided per number of copies. Dots represent pooled data from 20 donors (n = 15 p55^+^ and n = 5 p34^+^), and data are retrieved from the single-cell TCR dataset.

Besides the identification of TCRαβ V and J fragments from the bulk RNA-seq data, we also looked at the conservation of paired V and J fragments using the sequencing data on single-cell-sorted TM^+^ T cells for p34 and p55 and CD8^+^ TM^−^ T cells. Analysis at the single-cell level does not only permit analysis of the V-J fragment usage but can also access α and β pairing and will permit selection of TCRs for downstream analysis. Varying per donor, between 4 and 64 V-J pairs were identified in the single-cell dataset, which indicates that multiple different V-J pairs can bind to the same HLA-E/peptide complex. The V-J pairing was diverse for CD8^+^ TM^−^ T cells as well. Examples of two individual donors are shown in Figure 2A. The median CDR3 length in all HLA-E p55 and p34 TM^+^-sorted T cells was 41 nucleotides (1.0 SD) in the TCRα chain, whereas the CDR3β was slightly longer with a median length of 44 nucleotides (1,4 SD) (Figure 2B). The V(D)J-insert in the TCRβ chain in the single-cell-sorted TM^+^ T cells was also longer (median 12 ± 1.5 SD) than in the TCRα chain (median 4 ± 0.6 SD). Paired V-J analysis on the sorted single HLA-E p55 and p34 TM^+^ T cells per donor also revealed that the number of unique V-J pairs for both chains increased proportionally, indicating equal variation in the α and β chains (Figure 2C). HLA-E allele expression (i.e., ^∗^01:01 homozygous or ^∗^0101:^∗^0103 heterozygous) did not influence α- or β-chain variability, indicating no effect of genotype. Duplicate V-J pairings showed slight variations for some donors, which suggests that some V-J combinations are more preferred than others (Figure 2C). The number of CDR3 clonotypes with multiple copies was calculated per donor (Figure 2D) and most TCRα- and β-CDR3s were present as a single copy, suggesting diversity of V(D)J pairs in HLA-E p34 and p55 TM^+^ TCRs within and between donors, including for CD8^+^ TM^−^ TCRs. Similar clonotype analysis and comparisons could not be performed on single-cell-sorted HLA-E UL40 TM^+^ T cells because of the limited number of donors with a detectable TM^+^ population.

### Large variation in the number and usage of Vαβ and Jαβ fragments in single-cell-sorted HLA-E *Mtb* TM^+^ TCRs

**Figure 3:**
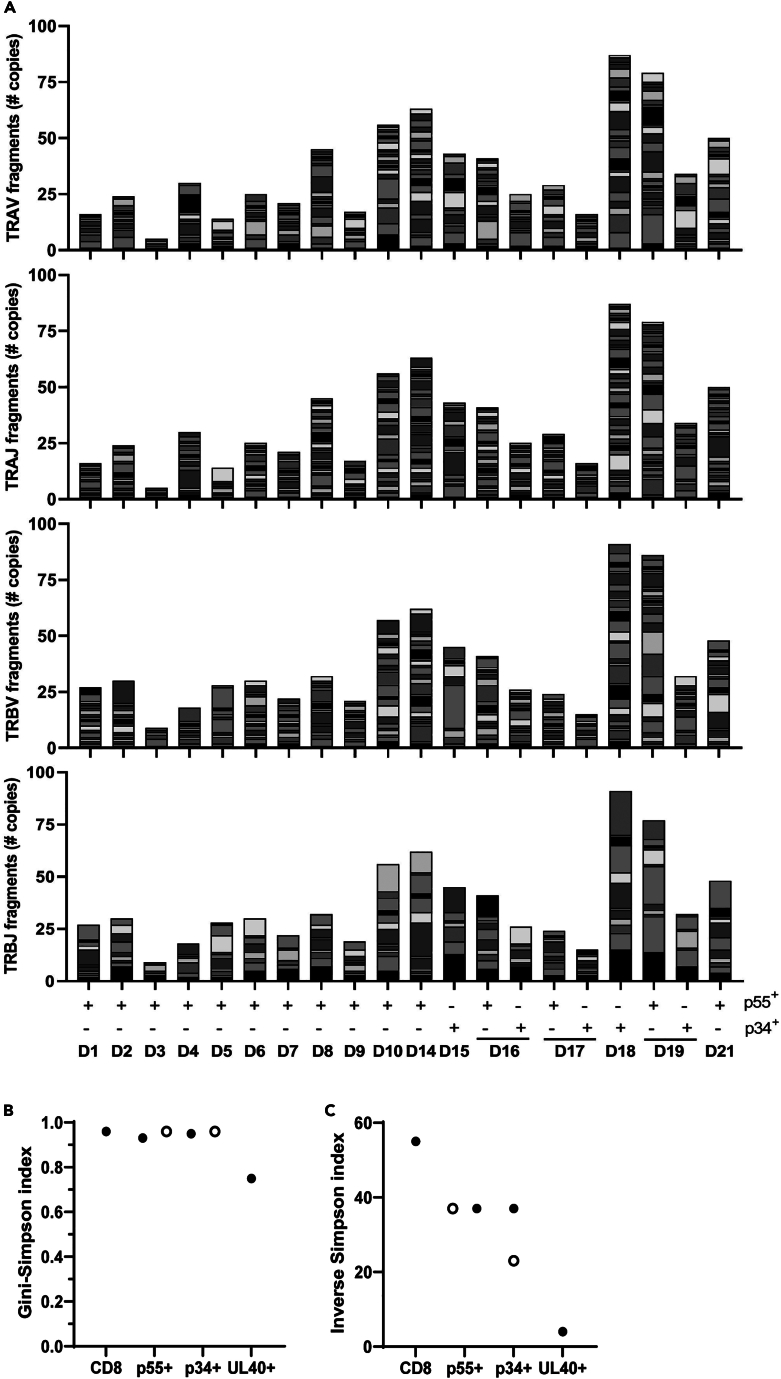
V and J fragment usage and frequency in p55 and p34 TM^+^ T cell populations (A) Absolute number of copies per TRAV, TRAJ, TRBV, and TRBJ fragment (top to bottom) in each of the 17 donors for either p55^+^ or p34^+^ TM^+^ TCRs. Data was obtained from the single-cell TCR sequencing dataset. Bar sizes correlate to the absolute number of copies per fragment. Data are shown per donor and per population. (B) CDR3 sequence diversity represented with the Gini-Simpson index. Gini-Simpson score of 1 indicates infinite clonal variation. Open dots represent the single-cell TCR dataset (n = 15 p55^+^, n = 5 p34^+^); closed dots represent the RNA-sequencing TCR dataset (n = 17 CD8^+^ TM^−^, n = 16 p55^+^, n = 2 p34^+^, n = 2 UL40^+^). (C) CDR3 sequence diversity represented with the inverse Simpson index. An inverse Simpson index of 1 defines a homogeneous TCR population with only 1 clonotype, and this index increases in parallel to the diversity to a limit related to the total number of reads.

Figure 3A shows the copy number of single Vα, Vβ and Jα, Jβ fragments in 17 donors extracted from the single-cell data. Stacked bars show the diversity as well as inter-donor variations in the number and usage of V and J fragments. To further substantiate TCR diversity quantitatively instead of numerically, we calculated the Gini-Simpson index as well as the inverse Simpson index on the single-cell data and RNA-seq data combined. The Gini-Simpson index indicates the probability that two randomly selected TCRs from the same population have a different clonotype. The index value can be between 0 and 1, where 1 represents a TCR repertoire of infinite diversity (i.e., polyclonal repertoire) and 0 indicates a monoclonal repertoire. CD8^+^ TM^−^, p34 TM^+^, and p55 TM^+^ TCRs had a Gini-Simpson index close to 1, indicating a more polyclonal TCR repertoire, whereas UL40 TM^+^ TCRs had a lower index (0.73), suggesting a relatively more monoclonal population (Figure 3B). The inverse Simpson index represents the inverse fraction of a particular clonal type relative to the total number of identified clonal types. An increasingly higher number represents a polyclonal repertoire, as shown for the total CD8^+^ TM^−^ T cells (Figure 3C). Compared with the total CD8^+^ TM^−^ T cells, p34 and p55 TM^+^ T cells had a lower index, suggesting a less polyclonal population compared with CD8^+^ TM^−^ T cells but still far from monoclonal. However, UL40 TM^+^ TCRs had a low index, close to 0, indicating an almost monoclonal population, which confirms the low number of TCR clonotypes detected in the bulk RNA-seq data for UL40 TM^+^ TCRs in the two donors (Figures 1A–1C).

### HLA-E *Mtb* TM^+^ CD8^+^ T cells have a different gene expression profile compared with CD8^+^ TM^−^ T cells

Next to extraction of TCR sequences from the bulk RNA-seq data, we also analyzed and compared the gene expression profile of HLA-E p55 TM^+^ CD8^+^ T cells with CD8^+^ TM^−^ T cells. We did not include HLA-E p34 TM^+^ CD8^+^ T cells because we could only sort p34 TM^+^ CD8^+^ T cells from two donors. This comparison showed that 276 genes were differentially expressed (DE), of which only one gene, i.e., PLXNB1, was significantly downregulated on p55 TM^+^ CD8^+^ T cells (Figures S2A and S2B). Plexin B1 is a receptor for Semaphorin 4. Signaling via Plexin B1 activates cell migration of neuronal, epithelial, and tumor cells, including angiogenesis. The function of PLXNB1 on T cells has not been unraveled yet. Principal-component analysis on all genes further substantiated the differences between total CD8^+^ TM^−^ and p55 HLA-E TM^+^ T cells and confirmed that the two T cell populations can be discriminated based on the gene expression profile (Figure S4A). Gene Ontology (GO) enrichment analysis revealed that the functional phenotype of p55 HLA-E TM^+^ T cells was also different compared to bulk CD8^+^ TM^−^ T cells. The top hits from the GO enrichment analysis were neutrophil activation, positive regulation of cytokine production + peptidyl-tyrosine phosphorylation, and NF-kB activation (Figure S3). Apart from neutrophil activation, these enriched functions point toward a metabolically activated phenotype of the p55 HLA-E TM^+^ T cells, although we cannot deduce their differentiation phenotype.

The top 25 genes with the highest Log2FoldChange differential expression between total CD8^+^ TM^−^ T cells and p55 HLA-E TM^+^ CD8^+^ T cells were analyzed further to identify if the proteins were specific for *Mtb* HLA-E TM^+^ CD8^+^ T cells (Figure S2C). These top 25 genes are involved in processes related to the Golgi apparatus and endoplasmic reticulum (ER), signaling transduction pathways (e.g., transforming growth factor β [TGF-β] and Wnt signaling), metabolic processes (e.g., ubiquitin ligases), and cell growth. RNA-seq read counts of these 25 genes were increased in the p55 HLA-E TM^+^-sorted population relative to the total CD8^+^ TM^−^ T cell pool (Figure S2C). We next validated if the proteins were also upregulated on p55 HLA-E TM^+^ T cells using cell surface staining in combination with TM staining. We selected genes for this validation if the gene was expressed in five donors or more, was an established cell surface protein, and had an average read count above 50. Eight of twenty-five genes fulfilled the criteria for protein-level validation but due to unavailability of flow cytometry antibodies for STAB1 and AQP9, only CD300b, TLR4, CCR2, THBD, FZD1, and WLS could be validated on 11 independent donors from the same TB endemic cohort (which were not included in the sequencing data) (Figure S4B). CD300b, TLR4, CCR2, THBD, and FZD1 were, in agreement with the RNA-seq data, significantly more expressed on the cell surface of p55 HLA-E TM^+^-sorted T cells, whereas only WLS, a carrier protein involved in Wnt signaling, was not (Figure S4C).

### Identification of TCRs with high peptide specificity using GLIPH2 clustering

**Figure 4:**
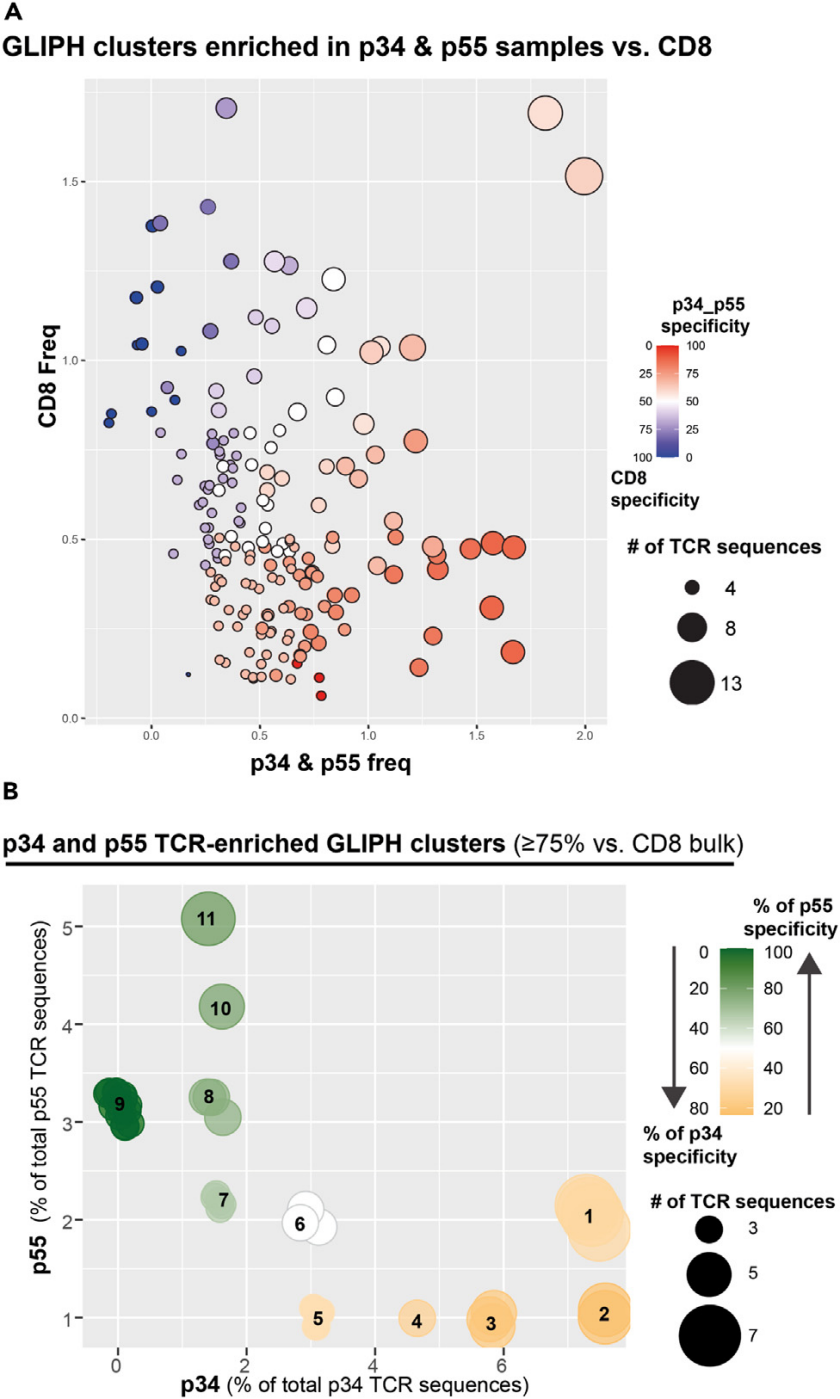
GLIPH2 analysis to identify TCRβ sequences enriched for either the p34 or p55 peptide (A) One hundred seventy-nine GLIPH2 clusters were identified in the bulk and single-cell sequence data of TCRs sorted with p34 or p55 TMs after filtering to exclude GLIPH2 clusters containing sequences from less than three donors. Plot shows clusters that are enriched for p34/p55 specificity in red or for bulk CD8^+^ TM^−^ in blue (scale on right side). Size of cluster represents the number of TCR sequences in that cluster (increasingly larger dot = increasingly higher number of TCR sequences). (B) Forty-one GLIPH2 clusters from the 179 clusters shown in (A) remain after selecting for clusters with enrichment for p34 (yellow) or p55 (green) over bulk CD8^+^ TM^−^ (scale on right side). These clusters were grouped into 11 categories containing overlapping GLIPH2 motifs. Size of cluster represents the number of TCR sequences in that cluster (increasingly larger dot = increasingly higher number of TCR sequences).

We next wanted to functionally validate HLA-E TM^+^ TCRs that were common in the single-cell TCR-seq data. To identify these common HLA-E TM^+^ TCR sequences, we applied the GLIPH2 cluster^30^^,^^31^ algorithm on TCR sequences identified from the single-cell TCR-seq data and bulk RNA-seq data combined. GLIPH2 clusters TCRβ sequences based on short similarity regions (enriched motifs) in the CDR3β region that are determined from the total TCRβ dataset via sequence alignment. The total input dataset included 3,211 TCRβ sequences. These sequences included TCRβs that could bind to HLA-E p34 and p55 TMs, and CD8^+^ TM^−^ TCRβs. Initially, 527 GLIPH2 clusters containing 1,489 TCRβ sequences were identified, which were filtered to exclude clusters containing sequences from less than three donors, resulting in 179 GLIPH2 clusters (Figure 4A). From these clusters, we selected clusters that contained TCRβ sequences with a higher p34/p55 TM specificity compared to bulk CD8^+^ TM^−^ TCRs. This selection resulted in 41 GLIPH2 clusters (Figure 4B). These clusters were then further separated for specificity to p34 or p55 TMs, which revealed that these clusters could be grouped into 11 categories (Figure 4B). We ultimately selected TCRβ sequences from these 11 categories using the following approach:

(1) We grouped all CDR3β sequences per category from the bulk and single-cell TCR-seq data that have the GLIPH2 motifs shown in Table 1.

**Table 1:** GLIPH2 identified motifs in the 11 categories

1	2	3	4	5	6	7	8	9	10	11
SMETRI%E	TRIG	SEEN%E	SDEAEH%ET	CASSDISGREQYF	SLL%GWAYNE	CASSLYRQVQYF	SAGQ%NTE	CASSAWGLQETQYF	SLGN%	S%GQKNTE
SMET	RIGE	DEAE		CAWAPGYTF	SLA%YNE	SLSGSD%E	S%GGNTE	SIDLFWVN%E		
METR		SDEA		SIV%TDT	WAYN	SL%GYE	DLF	SLAVSS%NE		
ETRI						SL%ET		SST%NTE		
						LNRD		SLG%SYE		
								SL%VSSYNE		
								SL%AGGTE		
								S%SSYNE		
								S%RGGTE		
								S%PNSP		
								S%GTSGAYE		
								S%GANE		
								EGG%SYE		
								%YGQDYE		
								%LGVSGANV
(2) We selected CDR3β sequences with more than 1 GLIPH2 motif.
(3) We selected CDR3β sequences that were derived from the single-cell TCR-seq dataset to find the pairing TCRα.
(4) We selected CDR3β sequence with restricted VJ gene variation.
(5) If possible, we preferred CDR3β sequences that were found in multiple donors or that had GLIPH2 motifs from >1 categories (i.e., the CDR3β sequence was found in multiple categories).
(6) We only selected CDR3β sequences from categories that had the highest percentage specificity for either p34 or p55 found in our analysis to prevent the inclusion of possibly peptide a-specific TCRs.

This approach ultimately led to the selection of 16 TCRβ and paired TCRα sequences from categories 1, 2, 3, 4, 8, 9, and 11, with a balanced distribution between p34 and p55 specificity (Tables 2A and 2B). This table also shows which GLIPH2 motifs are present in the selected TCRβs, indicated by the category number in the first column. For instance, TCR 13 contains all motifs present in categories 1 and 2.

**Table 2:** TCRαβ sequences selected with GLIPH2 analysis for transduction in Jurkat cells and primary CD3^+^ T cells

GLIPH2 category for p34	TCR#	Donor	CDR3β sequence	Vβ fragment	Jβ fragment	CDR3α sequence	Vα fragment	Jα fragment
**A**								

1 and 2	13	D15	CASSMETRIGEQYF	TRBV19	TRBJ2-7	CAEISDSSASKIIF	TRAV5	TRAJ3
3 and 4	14	D15	CASSDEAEHLETQYF	TRBV2	TRBJ2-5	CALPHYGGSQGNLIF	TRAV1-1	TRAJ42
3 and 4	15	D15	CASSDEAEHQETQYF	TRBV2	TRBJ2-5	CALPHYGGSQGNLIF	TRAV1-1	TRAJ42
3	16	D15	CASSDEAELLETQYF	TRBV2	TRBJ2-5	CALPHYGGSQGNLIF	TRAV1-1	TRAJ42
3	18	D15	CASSEENTEAFF	TRBV19	TRBJ1-1	CGYRSALSDGNKLVF	TRAV38-2DV8	TRAJ47
8 and 11	19	D19	CASSAGQKNTEAFF	TRBV6-5	TRBJ1-1	CILSIDTGNQFYF	TRAV26-2	TRAJ49

GLIPH2 category for p55	TCR#	Donor	CDR3β sequence	Vβ fragment	Jβ fragment	CDR3α sequence	Vα fragment	Jα fragment
**B**								

n.a.	8	D21	CASSPGSNQPQHF	TRBV4-2	TRBJ1-5	CAVINAGNMLTF	TRAV3	TRAJ39
3	17	D15	CASSEENTEAFF	TRBV19	TRBJ1-1	CAYRSALSDGNKLVF	TRAV38-2DV8	TRAJ47
8 and 11	20	D19	CASSAGQKNTEAFF	TRBV6-5	TRBJ1-1	CILSIDTGNQFYF	TRAV26-2	TRAJ49
8	21	D8	CASSIDLFWVNTEAFF	TRBV19	TRBJ1-1	CAGAPTHSWGKLQF	TRAV27	TRAJ24
8 and 11	22	D7	CASSSGQKNTEAFF	TRBV6-5	TRBJ1-1	CILSYDTGNQFYF	TRAV26-2	TRAJ49
9	24	D10	CASSLGVSGANVLTF	TRBV28	TRBJ2-6	CAMSEGDTGTASKLTF	TRAV14DV4	TRAJ44
9	25	D10	CASSLGVSGANVLTF	TRBV28	TRBJ2-6	CAMRDDDTGTASKLTF	TRAV14DV4	TRAJ44
9	26	D21	CASSYGQDYEQYF	TRBV6-5	TRBJ2-7	CAVSGFKITGGGNKLTF	TRAV8-6	TRAJ10
9	27	D6	CASSYGTSGAYEQYF	TRBV6-2	TRBJ2-7	CAMREGLRNQGGKLIF	TRAV14DV4	TRAJ23
9	28	D10	CASSLAVSSYNEQFF	TRBV11-1	TRBJ2-1	CAAQDNYGQNFVF	TRAV21	TRAJ26

(A) p34 TCR sequences. (B) p55 TCR sequences. TCR 8 specific for p55 was selected based on the high clonal number and not on the GLIPH2 results.

### Functional validation of GLIPH2-selected TCRαβ sequences

**Figure 5:**
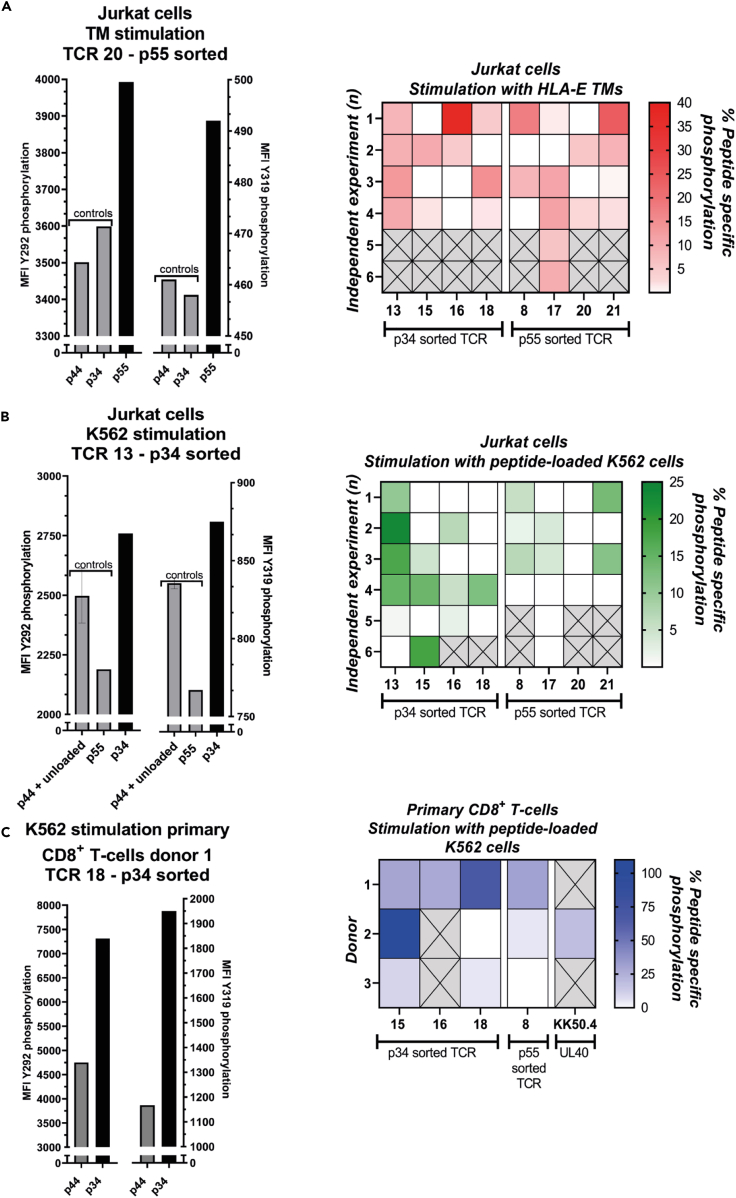
Zap70 phosphorylation of TCR-transduced Jurkat cells and primary CD8^+^ T cells sorted with TMs folded with p34 or p55 (A) Left: Representative MFI data after stimulating TCR 20 transduced Jurkat cells with HLA-E TMs. Graph shows the control conditions, including the heterologous peptide, in grey and peptide specific condition in black. Right: Percentage peptide specific Zap70 phosphorylation of 8 TCRs in Jurkat cells; 4 sorted with p34 TMs and 4 sorted with p55 TMs, after stimulation with HLA-E TMs loaded with control and specific peptides.Left: Representative MFI data after stimulating TCR 20 transduced Jurkat cells with HLA-E TMs. Graph shows the control conditions, including the heterologous peptide, in grey and peptide specific condition in black. Right: Percentage peptide specific Zap70 phosphorylation of 8 TCRs in Jurkat cells; 4 sorted with p34 TMs and 4 sorted with p55 TMs, after stimulation with HLA-E TMs loaded with control and specific peptides. (B) Left: Representative MFI data after stimulating TCR 13 transduced Jurkat cells with peptide-loaded K562 HLA-E cells. Graph shows the control conditions, including the heterologous peptide, in grey and peptide specific condition in black. Right: Percentage peptide specific Zap70 phosphorylation of 8 TCRs in Jurkat cells; 4 sorted with p34 TMs and 4 sorted with p55 TMs, after stimulation with K562 HLA-E cells loaded with control and specific peptides. (C) Left: Representative MFI data after stimulating TCR 18 transduced primary CD8^+^ T cells with peptide-loaded K562 HLA-E cells. Graph shows the control conditions in grey and peptide specific condition in black. Peptide specific phosphorylation in primary CD8^+^ T cells relative to the control peptide was determined via gating on CD8^+^ T cells within the CD3^+^ T cell population. Right: Percentage peptide specific Zap70 phosphorylation in transduced primary CD3^+^ T cells derived from 3 donors after stimulation with K562 HLA-E cells loaded with control and specific peptides. Each box in A-C represents an independent experiment for each TCR as indicated on the X-axis. The percentage representing each box was the highest peptide-specific phosphorylation site for that experiment and TCR. The independent experiment numbers on the Y-axis do not correlate and should be considered as individual experiments. A cross implies that there was no data for this TCR due to unsuccessful transduction into primary CD3^+^ T cells or because the TCR Jurkat line was not tested for that repetition (n).

The 16 TCRαβ sequences shown in Tables 2A and 2B were transduced into Jurkat cells that did not express endogenous TCR sequences or into isolated primary CD3^+^ T cells derived from three healthy buffy coats.^32^ HLA-E-restricted T cells have an unorthodox cytokine profile, with the capacity to secrete both Th1 and Th2 cytokines. Functional readout methods based on cytokine secretion, such as ELISpot or ICS that are commonly used for classical HLA-I T cells, are difficult, because of the overall low cytokine levels and the variation in the specific cytokines secreted by specific HLA-E T cells. We therefore used Zap70 phosphorylation as a readout for early TCR activation, which is a cytokine-independent method. Based on the transduction efficiencies, we selected 4 p34 HLA-E TM^+^-sorted TCRs and 4 p55 HLA-E TM^+^-sorted TCRs transduced in Jurkats and 3 p34 HLA-E TM^+^-sorted TCRs and 1 p55 HLA-E TM^+^-sorted TCR transduced in primary CD8^+^ T cells (after gating on the CD8^+^ T cell population within the transduced CD3^+^ T cell population) to validate their peptide specificity via measuring Zap70 activation after stimulation with peptide-loaded K562 cells expressing HLA-E^∗^01:03 or with HLA-E^∗^01:03 TMs loaded with *Mtb* peptides (for Jurkat cells only). Zap70 is activated early after TCR signaling via phosphorylation of tyrosine (Y) at positions 292 and 319, among other sites, referred to as Y292 and Y319. In one of our previous studies, Zap70 phosphorylation was a good indicator for activation of HLA-E-specific T cell clones.^12^ We determined the level of phosphorylation at Y292 and Y319 and calculated the percentage of peptide-specific phosphorylation at Y292 and Y319 relative to the mean fluorescent intensity (MFI) of the control peptide (p44) and, in the case of K562 stimulation, unloaded condition. The left panels in Figures 5A–5C show representative examples of the MFI of phosphorylated Y292 and Y319 for the peptide-specific condition and the control conditions in TCR-transduced Jurkat cells and TCR-transduced primary CD8^+^ T cells after stimulation with peptide-loaded K562 cells or HLA-E TMs (Jurkat cells only). Representative gating strategies are shown in Figure S5 for both primary CD8^+^ T cells and Jurkat cells. We selected the highest phosphorylation site per TCR and per independent experiment/donor after peptide stimulation and summarized these results in Figures 5A–5C right panels for both transduced Jurkat cells and primary CD8^+^ T cells per stimulus. The *Mtb* peptide that was used for sorting T cells is shown below the heatmaps. These results show that most TCRs could be activated in an HLA-E/peptide-specific manner with one of the stimuli in both Jurkat cells and primary CD8^+^ T cells. The shift in Zap70 phosphorylation was not very strong, likely reflecting weak interaction between the TCR and the HLA-E peptide complex. There was inter-experimental variation as well as variation between TCRs in the maximal signal of Zap70 activation after peptide specific stimulation but overall TCRs showed repeatedly a positive signal above background. Most *Mtb* HLA-E TM^+^-sorted TCRs, however, had a comparable signaling capacity as the published HLA-E-restricted TCR KK50.4, which recognizes the UL40-derived peptide with high affinity, at least for primary CD8^+^ T cells (Figure 5C, right).^33^

Although the transduction efficiency of the TCRs in primary CD8^+^ T cells was similar between donors, there was variation between donors in the percentage phosphorylation that could be achieved upon peptide stimulation for the same TCR suggesting a donor-dependent effect (e.g., TCR 8 and 18). Likewise, the percentage phosphorylation upon peptide stimulation in TCR-transduced Jurkat cells showed variation between experiments and between stimuli (Figures 5A and 5B). In general, HLA-E TMs induced a higher level of phosphorylation compared with peptide-loaded K562 cells expressing HLA-E. Nevertheless, the overall percentage of phosphorylation upon peptide stimulation was higher in primary CD8^+^ T cells than in Jurkat cells.

## Discussion

We describe, what is to our knowledge, the first elaborate in-depth TCR repertoire analysis of CD8^+^ T cells binding to two *Mtb* peptides presented in HLA-E^∗^01:03. Our TCR sequencing results reveal that TCRs sorted based on binding to HLA-E TMs loaded with p55 and p34 have a heterogeneous repertoire within and between individuals. Several V and J gene segments in both TCRα and β chains were identified on sorted HLA-E TM^+^ p55 or p34 TCRs. TCRs showed limited clonal expansions, as confirmed quantitively with a close-to-1 Gini-Simpson index, revealing a TCR Vαβ profile that was almost comparable with that of bulk CD8^+^ TM^−^ T cells. However, the number of *Mtb*-peptide-specific (functional) TCRs is potentially overestimated as TM staining was used to identify *Mtb* HLA-E-specific TCRs. This was also found previously and might be an inherent problem of using TMs to identify peptide-specific functional TCRs.^34^ Despite this limitation, our TCR sequencing results suggest that HLA-E TCRs are less conserved compared to previously described other DURTs.^35^

We also showed that HLA-E *Mtb* TM^+^ CD8^+^-sorted T cells have a distinctive gene expression profile compared to bulk CD8^+^ TM^−^ T cells, showing upregulation of all but one of the 275 differentially expressed genes in HLA-E *Mtb* TM^+^ CD8^+^ T cells, suggesting a more (transcriptionally) activated state of HLA-E *Mtb* TM^+^ CD8^+^ T cells compared to bulk CD8^+^ TM^−^ T cells. Differences between bulk CD8^+^ TM^−^ T cells and *Mtb* HLA-E TM^+^-sorted CD8^+^ T cells were also found previously in cellular assays, showing that TM^+^ CD8^+^ T cells had a higher production of interferon gamma (IFN-γ), interleukin-10 (IL-10), IL-4, and granulysin, suggesting that *Mtb* HLA-E TM^+^ T cells are transcriptionally and functionally distinct compared to bulk CD8^+^ TM^−^ T cells.^13^ The activated state of *Mtb* HLA-E TM^+^ T cells compared to bulk CD8^+^ TM^−^ T cells could have been induced upon TM binding. However, the read count of several genes known to be upregulated upon T cell activation (e.g., granulysin, IFN-γ, IL-2) were not increased in the sorted HLA-E TM^+^ population compared to the sorted HLA-E TM^−^ population.

The large variation of TCRs binding HLA-E/*Mtb* TM complexes is remarkable, suggesting that HLA-E-restricted T cells are more similar to conventional MHC-restricted T cells compared to DURTs in terms of their TCR profile.^24^ The diversity of classical TCRs recognizing *Mtb* was illustrated before in two recent studies that performed TCR repertoire analysis on sorted activated (i.e., CD154- and CD69-positive) CD4^+^ T cells obtained from PBMCs of *Mtb*-infected individuals briefly stimulated with *Mtb* lysate.^30^^,^^36^ Similar to our results on *Mtb* TM-sorted CD8^+^ HLA-E T cells, GLIPH2 analysis in both studies identified various unique TCRβ sequences that could be clustered into >100 unique TCR specificity groups based on shared CDR3β motifs identified by GLIPH2. Unlike our approach, these studies used GLIPH2 to determine the cognate peptide and HLA-II allele restriction of the identified TCR clusters to curate their approach as *Mtb* lysate contains many epitopes. The TCR repertoire of these conventional CD4^+^ T cells recognizing various HLA-II alleles seems to be comparable with the diversity we have identified for CD8^+^ HLA-E *Mtb* TM^+^ T cells. In contrast, a recent study revealed a restricted TCR repertoire and invariant TCRα usage in MAIT cells, which also recognize a monomorphic antigen presentation molecule and are well known as DURTs.^35^ These MAIT cells were sorted from PBMCs stimulated with *Mtb* lysate on the basis of CD161 expression.^35^ These studies, suggest that *Mtb* can be recognized by a potentially diverse TCR repertoire in the context of HLA-E and further suggest that HLA-E T cells are more similar to conventional T cells than other DURTs.

Similar to our findings for *Mtb*, a diverse TCR profile was identified before for 13 HLA-E TCRs recognizing the HIV-derived RL9HIV peptide presented in HLA-E, which were obtained from one donor and 13 HLA-E TCRs in two donors recognizing Sars-CoV-2 peptides.^27^^,^^28^ These TCRs revealed diverse V-J pairs in both α and β chains, including heterogeneity in the CDR3 region. In contrast, we observed a more clonally expanded TCR population in two donors, particularly in the TRBV region, within the CD8^+^ T cell population recognizing the UL40-derived HLA-E-specific peptide from CMV. In one donor, the TRBV20-1 fragment was dominant, whereas in the other donor TRBV5-1 was dominant. Similar monoclonal expansions and donor dependency were described before in HLA-E T cells from four donors recognizing various epitopes derived from the UL40 protein. Cytolytic T cell clones derived from these donors generated using a limited dilution culturing system expressed either TRBV16, 9, 22, and 5-1 with a percentage ranging from 60% to 80%.^15^^,^^26^ However, this seems rather unique for UL40 epitopes presented in HLA-E, as in that specific case also the genotype of HLA class Ia alleles restricts the induction of HLA-E-specific responses. Even though a more restricted TCR repertoire could indicate memory formation, it is known that DURTs (although not evaluated for HLA-E) due to their well-described effector and innate-like character generally do not differentiate to a memory phenotype, as was shown before in the context of BCG vaccination.^37^

The lack of genetic diversity of HLA-E TCRs recognizing UL40-derived epitopes compared with TCRs recognizing *Mtb* or other pathogen-derived peptides could be explained by the sequence similarities between UL40 epitopes and HLA-Ia leader-sequence-derived peptides, which CMV exploits to avoid lysis of the infected cell. It is conceivable that most of the higher affinity TCRs recognizing UL40 peptides identical to self-peptides undergo negative selection in the thymus to avoid the development of self-reactive T cells. The limited number of UL40 TCRs that escape negative selection possibly share the same or similar sequences, leading to a more conserved population. For other pathogens, as we have shown previously for *Mtb*, various epitopes with sequences divergent from self-peptides can be presented by HLA-E; thymic selection likely permits the generation of a diverse TCR repertoire.^17^ The recognition of HLA-E/*Mtb* peptide complexes by multiple TCRαβ combinations is possibly also beneficial for vaccination with HLA-E peptides as the HLA-E/peptide complex can be recognized by a larger pool of TCRs. In that line, it is interesting to know if a diverse TCR repertoire remains after vaccination with HLA-E peptides as a primary response.

We selected HLA-E *Mtb* TM^+^ TCRs for functional validation based on the GLIPH2 analysis performed on the bulk RNA-seq and single-cell TCR sequence data. The activation of these TCRs using Zap70 phosphorylation of Y292 and Y319 as a readout after K562 and TM stimulation showed variable results between experiments and TCRs sorted for the same *Mtb* peptide. Some TCRs displayed relatively clear Zap70 signals; however, several TCRs had limited Zap70 activation after peptide stimulation. Possibly, this is the result of low affinity interactions that are difficult to distinguish from a-specific interactions, therefore not ruling out a degree of a-specificity. A recent study on HLA-E TCRs from T cell clones recognizing SARS-CoV-2-derived peptides also found weak functional T cell responses upon peptide stimulation and dim TM staining that could be explained by the low TCR affinity for HLA-E.^28^ Besides this, the requirements for HLA-E TCR activation (i.e., co-stimulation signals and cytokines) are not known, and possibly, experiments to induce TCR activation were performed under suboptimal conditions.

Altogether, this study is another step forward in the understanding of the diverse and complex HLA-E-*Mtb*-restricted immunity, which is crucial in the advancement of TB vaccine design.

### Limitations of the study

There are some aspects in our studies that could be improved in future work using latest state-of-the-art methods currently available. First, the validity of our sorting and RNA-seq on sorted CD8^+^ HLA-E TM^+^ T cells could have been improved with the use of dual TM labeling methods followed directly by single-cell RNA-seq. However, to prevent sorting of a-specific HLA-E TM^+^ cells, we stained with two unrelated HLA-E TMs attached to different fluorochromes and sorted only the single positive HLA-E TM^+^ population. With this setup also cells with a-specific HLA-E TM staining would not be included in the analysis. Second, our statements on p34 and p55 HLA-E TM^+^-specific gene signatures and TCR profiles based on the transcriptomic data could have been strengthened by comparing and contrasting to other ([non-]classical) T cell subsets, such as HLA-E-restricted CMV-specific T cell responses and HLA-A2-restricted responses, as well as by including several of our recently predicted *Mtb*-derived HLA-E-specific peptides. Although we intended to have a CMV-specific control population, the individuals in the studied cohort did not have detectable responses toward the HLA-E peptide from CMV that we included as a control for our sequencing analysis. Third, we included individuals from one geographical location, namely South-Africa, but the frequency of circulating HLA-E T cells and the capacity to recognize HLA-E-specific *Mtb* peptides could depend on the geographical location and TB burden. In addition to this point, we did not perform sex-related analyses. However, the proportion of male to female in our cohort was almost 1:1 (10 males and 11 females), which suggests that the data are representative for both sexes. Lastly, we only included two *Mtb*-specific HLA-E-restricted epitopes in this study. Including more HLA-E-specific *Mtb* epitopes might provide a broader overview of the HLA-E *Mtb* T cell repertoire and would increase the chance of detecting HLA-E/*Mtb* T cells in the circulation. Due to the limited PBMC material available for each individual, analyses had to be prioritized and were necessarily limited.

## STAR★Methods

### Key resources table

**Table:** 

REAGENT or RESOURCE	SOURCE	IDENTIFIER
**Antibodies**		

mouse anti-human CD94 unconjugated monoclonal antibody clone HP-3D9	BD Biosciences	Cat. no: 555887; RRID: AB_396199
mouse anti-human CD4 clone OKT4 BV650	BD Bioscience	Cat. no: 750989; RRID: AB_2875058
mouse anti-human CD8 clone HIT8a FITC	BD Bioscience	Cat. no: 555634; RRID: AB_395996
mouse anti-human CD3 clone UCHT1 BV510	Biolegend	Cat. no: 300448; RRID: AB_2563467
hamster anti-mouse constant TCRβ clone H57-597 APC-Cy7	BD Bioscience	Cat. no: 560656; RRID: AB_1727574
mouse anti-human Zap70 (pY319)/Syk (Y352) clone 17A/P-ZAP70 PE-Cy7	BD Bioscience	Cat. no: 561458; RRID: AB_10696417
mouse anti-human Zap70 (pY292) clone J34-602 PE	BD Bioscience	Cat. no: 558510; RRID: AB_647236

**Biological samples**		

Human peripheral blood mononuclear cells	Participants in this study	na
Buffy coats for CD3^+^ T cell isolation	Sanquin blood transfusion services	na

**Chemicals, peptides, and recombinant proteins**		

DNAse I	Calbiochem	Cat. no: 26-091-310MU
QIAzol Lysis Reagent	Qiagen	Cat. no: 79306
Proteinase K	Qiagen	Cat. no: 19131
Taq DNA polymerase	Qiagen	Cat. no: 201203
10× PCR Buffer	Qiagen	Cat. no: 201203
Deoxynucleotide triphosphate	Invitrogen	Cat. no: 10297018
Fugene HD transfection reagent	Promega	Cat. no: E2311
CoralLoad PCR Buffer	Qiagen	Cat. no: C28201225
ExoSAP-IT Express reagent	Applied Biosystems	Cat. no: 75001.1.ML
RetroNectin	TaKaRa Bio	Cat. no: T100B
CD3 MicroBeads human - lyophilized	Miltenyi	Cat. no:130-097-043
Recombinant human IL-2	Peprotech	Cat. no: 200-02
Purified Phytohemagglutinin (PHA)	Remel Europe Ltd.	Cat. no: HA16
BD Cytofix™ Fixation Buffer	BD Bioscience	Cat. no: 554655
BD Pharmingen Stain Buffer (FBS)	BD Bioscience	Cat. no: 554656
BD Phosflow Perm Buffer III	BD Bioscience	Cat. no: 558050
p34 (VMTTVLATL)	Peptide 2.0	na
p55 (VMATRRNVL)	Peptide 2.0	na
p62 (RMPPLGHEL)	Peptide 2.0	na
p44 (RLPAKAPLL)	Peptide 2.0	na
CMV UL40 (VMAPRTLIL/VLAPRTLLL/VMAPRTLLL)	Peptide 2.0	na
HLA-E∗01:03 p34 TM	Produced in house	na
HLA-E∗01:03 p55 TM	Produced in house	na
HLA-E∗01:03 p62 TM	Produced in house	na
HLA-E∗01:03 CMV UL40 TM	Produced in house	na
HLA-E∗01:03 p44TM	Produced in house	na
Streptavidin APC	Thermo Fisher Scientific	Cat. no: S868
Streptavidin PE	Thermo Fisher Scientific	Cat. no: S866
Oxidized gluthathione	Sigma-Aldrich	Cat. no: G4376-10g
reduced gluthathione	Sigma-Aldrich	Cat. no: G4251-25g
Tris.HCl	Roche	Cat. no: 10-708-976-001
EDTA	GibcoBRL	Cat. no: 15576-028
L Arginine	Sigma-Aldrich	Cat. no: 10-229-4072
Complete protease inhibitors	Roche	Cat. no: 11-836-145-001
glycerol	Thermo Fisher Scientific	Cat. no: 17904
PBS	Fresenius	Cat. no: 362-3410
BirA	Produced in house	na
Na azide	In house pharmacy	na

**Critical commercial assays**		

R107G SNP rs1264457 Taqman SNP genotyping assay	Applied Biosystems	Cat. no: 4351379
miRNeasy Mini Kit	Qiagen	Cat. no: 217004
SMART-Seq version 4 Ultra Low Input RNA Kit	TaKaRa Bio	Cat. no: 634762
SuperScript VILO cDNA Synthesis Kit	Thermo Fisher Scientific	Cat. no: 11754050
LIVE/DEAD™ Fixable Violet Dead Cell Stain Kit	Invitrogen	Cat. no: L34955

**Deposited data**		

GEO	GSE236154	na
VDJdb	https://github.com/antigenomics/vdjdb-db/issues/351	na

**Experimental models: Cell lines**		

Human 293-based Phoenix GALV packaging cells	Dr. M. Heemskerk	na
CD8^+^ TCRko Jurkat E6.1 triple parameter reporter (TPR) cells	Dr. P. Steinberger	na
CD8^+^ TCRko Jurkat 76 triple parameter reporter (TPR) cells	Dr. M. Heemskerk	na
K562 HLA-E∗01:03 presenting cells	Dr. E. Weiss	na

**Oligonucleotides**		

Internal/External TRAV, TRBV, TRAC and TRBC primers	Wang et al. (2012)^38^	https://doi.org/10.1126/scitranslmed.3003647
TCR sequences specific for Mtb34 and Mtb55 HLA-E TM^+^ CD8^+^ T-cells	This manuscript Table 2	na
TCR sequences KK50.4 TCR	Hoare et al. (2006)^33^	https://doi.org/10.1038/ni1312

**Recombinant DNA**		

pMP71 flex retroviral vector	Linneman et al. (2013)^39^	https://doi.org/10.1038/nm.3359

**Software and algorithms**		

Geneious Prime version 2021.0	Geneious prime	
Biorender	Biorender	
GLIPH2	Algorithm published in Huang et al. (2020)^30^	https://doi.org/10.1038/s41587-020-0505-4
RStudio v1.2	R Foundation	
FACSDiva	BD Bioscience	
DESeq2 package	DESeq2	
MiXCR software version 2.1.9.	MiXCR	
VDJtools version 1.2.1	VDJtools	
tcR package	tcR	
GraphPad software version 8.0.1	Graphpad	
FlowJo v10.8.1 software	Treestar	

**Other**		

1% Paraformaldehyde	In house pharmacy	na
Fetal bovine serum	Capricorn	Cat. no: FBS-16A
Iscove’s Modified Dulbecco’s Medium (IMDM)	Gibco	Cat. no: 12440053
PBS	Fresenius Kabi Nederland B.V.	Cat. no: 16SD7331
Ficoll	In house pharmacy	na
Human serum albumin	Sanquin blood transfusion services	na
0.05% Trypsin-EDTA (1X)	Gibco	Cat. no: 25300-054
Bovine Serum Albumin Fraction V	Roche	Cat. no: 10735094001

### Resource availability

#### Lead contact

Further information and requests for resources and reagents should be directed to and will be fulfilled by the lead contact, Linda Voogd (l.voogd@lumc.nl).

#### Materials availability

This study did not generate new unique reagents.

#### Data and code availability

##### Data

Bulk RNA-seq data have been deposited at GEO and are publicly available as of the date of publication. Single VDJ and CDR3α/β sequences have been deposition at the VDJdb database and are publicly available as of the data of publication. Accession numbers are listed in the key resources table.

##### Code

This paper does not report original code.

##### Other items

Any additional information required to reanalyze the data reported in this paper is available from the lead contact upon request.

### Experimental model and study participant details

#### Recruited human participants

The South African Tuberculosis Vaccine Initiative (SATVI) at the University of Cape Town enrolled and followed healthy South African adolescents aged 14–18 years living in a highly TB endemic area in the Adolescent Cohort Study (ACS). The cohort consisted of 10 males and 11 females. Detailed information per participant, i.e., age, sex and ethnicity can be found in Table S1. Human participation to this study was in accordance with the principles of the Declaration of Helsinki. The study protocol was approved by the Research Ethics Committee of the University of Cape Town (protocol EPI-002-ZA, date of first approval 25 April 2005), and by the Independent Ethics Committee (IEC) of Aeras, the sponsor. All adolescent participants provided written assent, while parents or legal guardians provided informed written consent for participation in the study. All participants were BCG vaccinated at birth and HIV negative. Heparinized venous whole blood and QuantiFERON-TB Gold (QFT) In-Tube tests were obtained at regular intervals and disease progression was monitored.^40^^,^^41^ Our study included QFT positive as well as QFT negative participants, without any signs of active TB. Peripheral blood mononuclear cells (PBMCs) were isolated by Ficoll-density gradient centrifugation and cryopreserved in liquid nitrogen. Frozen samples were sent to the Department of Infectious Diseases at the Leiden University Medical Center, the Netherlands, where further experiments were performed.

#### Cell lines

Human 293-based Phoenix GALV packaging cells and CD8^+^ TCRko Jurkat 76 triple parameter reporter (TPR) cells were provided by Dr. M. Heemskerk. CD8^+^ TCRko Jurkat E6.1 triple parameter reporter (TPR) cells were provided by Dr. P. Steinberger. K562 HLA-E^∗^01:03 presenting cells were provided by Dr. E. Weiss. Unless stated otherwise, all cell lines were cultured in Iscove’s Modified Dulbecco’s Medium (IMDM, Invitrogen) supplemented with 10% Fetal Bovine Serum (FBS, Capricorn) and 1% penicillin/streptomycin (Gibco) at 37°C, 5% CO_2_.

### Method details

#### Flow cytometric sorting and analysis

PBMCs from 21 participants (Table S1) were thawed, spun down and resuspended in 200 μL Iscove’s modified Dulbecco’s medium (IMDM, Gibco Life technologies) and treated for 10 min at RT with 80.000 u/mL DNAse I (Calbiochem). After washing, the PBMCs were counted (CASY cell counter, Roche), resuspended in PBS +0.1% BSA and divided into 2-5x10^6^ cells per staining. The cells were then incubated for 10 min at RT with 10 μg/mL CD94 mAb (Clone HP-3D9, BD Bioscience) to block HLA-E tetramer (TM) binding to the CD94/NKG2A receptor complex.^13^ HLA-E TMs were produced as described previously and monomer folding was confirmed with mass spectrometry and staining to LILRB1 expressing cell lines.^17^^,^^18^^,^^29^ HLA-E TM staining was performed for 15 min at 37°C; containing an equal mix of HLA-E^∗^01:01 and HLA-E^∗^01:03 TMs, loaded with Mtb peptide #p55 (VMATRRNVL, 0.4 μg TM/10^6^ cells) conjugated with phycoerythrin (PE), Mtb peptide #p34 (VMTTVLATL, 0.8 μg TM/10^6^ cells) conjugated to allophycocyanin (APC) or loaded with CMV-UL40 glycoprotein derived peptide (VLAPRTLLL, 0.2 μg/10^6^ cells) conjugated with APC. PBMCs were stained with 2 different HLA-E TMs labeled with different fluorochromes. PBMCs were subsequently stained for surface expression of: CD3 (BV510, clone UCHT1, BD Biosciences), CD4 (BV650, clone OKT4, Biolegend), CD8 (FITC, clone HIT8a, BD Biosciences). Samples were stained in the dark for 30 min at 4°C, washed in PBS/0.1% BSA. A live/dead stain (Vivid fixable violet dye, Invitrogen) was performed on all samples according to the manufacturer’s protocol. Cells were then washed twice in PBS/0.1% BSA and resuspended to 5-10 x 10^6^ PBMCs/mL, strained through a 50 μM filter (CellTrics filters, Sysmex) and then acquired on a FACSAria-III cell sorter (BD Biosciences) using FACSDiva software (v8.0.2 BD Biosciences). We sorted only HLA-E TM^+^ cells if a single HLA-E TM^+^ population was visible. A minimum of 800 sorted cells were immediately lysed in in 250 μL QIAzol Lysis Reagent (Qiagen) and stored at −80°C for RNA sequencing. In addition, 100 single cells were sorted and collected in 96-well plates for identification of the single cell TCR sequences. The sorting populations of peptide specific TM-subsets were identified using the gating strategy as shown in Figure S1 and comprised the subsequent gating on the lymphocyte population according to the FCS vs. SSC plot, single cells, live cells, CD3^+^ cells, CD4^−^CD8^+^ cells and TM^+^ cells. The peptide specific populations sorted for TCR sequencing per donor are shown in Table S1.

#### HLA-E genotyping

HLA-E∗01:01 and/or ∗01:03 genotypes were determined with the R107G SNP rs1264457 Taqman SNP genotyping assay (Applied Biosystems). At least 10,000 PBMCs were frozen in MQ/1%BSA (Sigma-Aldrich) at −20°C. For analysis samples were thawed and incubated 5 min at 80°C, 10 min at 56°C with 1 μL Proteinase K (Qiagen) and 2 min at 80°C. Subsequently 25-μL qPCR reaction mixes were prepared containing 5–10 μL cell lysate, 12.5 μL Taqman Universal PCR 20× Mastermix (Thermo Fisher Scientific) and 0.15 μL Taqman probe mix rs1264457 (Applied Biosystems). Fluorescence was measured using a Quantstudio6 Flex (Qiagen) during qPCR conditions of 95°C for 5 min, 40 cycles of 95°C for 20 s, 52°C for 20 s, and 72°C for 45 s, followed by 1 cycle of 72°C for 7 min.

#### RNA sequencing

Following flow cytometric sorting, RNA was isolated from the QIAzol samples according to the manufacturer’s protocol using an miRNeasy Mini Kit (Qiagen) and eluted into DNA Lobind Eppendorf Tubes (VWR). RNA integrity and yield was tested on the Agilent 2100 Bioanalyzer. Quality control steps were included to determine RNA and RNA library quality and quantity. Samples that did not pass QC were excluded. RNA from samples of CD8^+^TMp55^+^ cell population paired with the donors’ CD8^+^ population from 16 donors were successfully acquired and suitable for RNA-seq. For 2 donors, we obtained RNA from CD8^+^TM CMV^+^ samples. For 1 donor we solely sequenced the CD8^+^ population as we could not acquire any TM^+^ cells. RNA was amplified to cDNA using the SMART-Seq version 4 Ultra Low Input RNA Kit for Sequencing (Takara Bio). The resulting cDNA was used to prepare a sequencing library. Libraries were sequenced on an Illumina HiSeq 2500 platform to acquire single-end reads of 50 base pairs in length (0.5 Gb/sample), resulting in approximately 17 million reads per sample of which 80% could be aligned against the human genome.

#### RNA sequencing analysis

Reads were aligned against the human reference genome: Homo sapiens GRCh38.91. Analysis of reads was performed in R (version 3.5.0, R Foundation, software: RStudio v1.2) using the DESeq2 package for the normalization and detection of differentially expressed genes. DESeq2 parameters used were: log2 foldchange (LFC) threshold = 1, read count >4 per gene, adjusted p value cut off = 0.05 and the DESeq2 multifactor design formula = ‘∼donor + cell population’. The cell populations compared were CD8^+^ and CD8^+^TMp55^+^. No samples were excluded. The DESeq2 model and analysis processes are described in Love et al. 2014.^42^ Briefly, the differential expression analysis in DESeq2 uses a generalized linear model using a negative binomial distribution with fitted mean and a gene-specific dispersion parameter. The raw RNA-seq data files are deposited at GEO (GEO accession number: GSE236154).

#### Single cell cDNA synthesis, multiplex nested PCR, and sanger sequencing

TM-specific sorted single cells were collected in PCR plates. cDNA was synthesized from these single cells with SuperScript VILO cDNA Synthesis Kit (Thermo Fisher Scientific) in 2.5-μL reaction mixes, each containing 0.25 μL of SuperScript III reverse transcriptase, 0.5 μL of 5× VILO reaction mix and 0.1% Triton X-100 (Sigma-Aldrich). Mixes were incubated at 25°C for 10 min, 42°C for 120 min, and 85°C for 5 min TCR transcripts from each cell were amplified by multiplex nested PCR in 25-μL reaction mixes containing 2.5 μL of cDNA. The external PCR was performed with 0.75 U of Taq DNA polymerase (Qiagen), 2.5 μL of 10× PCR Buffer (Qiagen) (containing KCl, (NH_4_)_2_SO_4_ and 15 mM MgCl_2_), 0.5 μL of 10 mM deoxynucleotide triphosphate (Invitrogen) and 2.5 pmol each of the external TRAV, TRBV, TRAC and TRBC primers. PCR conditions were 95°C for 5 min, followed by 35 cycles of 95°C for 20 s, 52°C for 20 s, and 72°C for 45 s, followed by 1 cycle of 72°C for 7 min. The internal PCR used CoralLoad PCR Buffer (Qiagen) containing two marker dyes for gel loading. 2.5 μL External PCR product served as template for two separate PCRs that incorporated either (a) internal sense TRAV primers and antisense TRAC primer or (b) internal sense TRBV primers and antisense TRBC primer. TCR segment-specific primers as published by Wang et al. 2012 were synthesized (Sigma-Aldrich), reconstituted and multiplexed to a concentration of 5 mM each for 40 external/internal pairs of sense TRAV, 28 sense TRBV, and 1 of each antisense TRAC and TRBC primers.^38^ Presence of internal PCR products was confirmed on a 2% agarose gel and aliquots (5 μL) of relevant samples were purified with 2 μL ExoSAP-IT Express reagent (Applied Biosystems) during 4 min at 37°C and 2 min at 80°C. If necessary, products with multiple bands on gel were run over a 1% low-melt agarose gel, single bands were selected and treated with the Wizard SV Gel and PCR Clean-Up System (Promega) according to manufacturer’s protocol. Transcripts were Sanger sequenced with 10 pmol of relevant internal antisense primer, either TRAC (TGTTGCTCTTGAAGTCCATAG) or TRBC (TTCTGATGGCTCAAACACAG), by BaseClear (Leiden, the Netherlands).

#### (paired) TCRα/β analysis

TCRαβ gene fragments from single cell Sanger sequencing, as well as TCRαβ gene fragments from bulk RNA-seq data, were identified -and paired for single cell data-using MiXCR software version 2.1.9. TCR gene segments were designated according to ImMunoGeneTics nomenclature (IMGT, http://www.imgt.org).^43^ CDR3α/β pairs displaying the same amino acid sequences and V/J gene usage were defined as clonotypes. Pairwise overlap circos plots were created and BasicStats calculated using VDJtools version 1.2.1 (VDJtools) and Gini-Simpson and inverse Simpson indices were calculated using the tcR package (tcR) in R version 3.5.1 (R).^44^^,^^45^ Graphs and statistics were performed using GraphPad software version 8.0.1 (Prism, La Jolla, USA). Obtained CDR3α/β amino acid sequences from bulk RNA-seq as well as from single cell TCR sequencing were used to construct clone network analysis with the GLIPH2 algorithm.^30^^,^^31^ TCR sequences are available at the VDJdb database: (https://github.com/antigenomics/vdjdb-db/issues/351). TCR sequences from sorted single cells with a high clonal number and high significance score as determined by GLIPH2 were selected for transductions and are shown in Tables 2A and 2B.

#### MP71flex vector design

The MP71flex retroviral vector was used to clone VDJ TCR segments of HLA-E TM specific TCRαβ chains. This vector contains several unique cut-sites which allows the exchange of variable TCRαβ domains into the vector and contains codon-optimized murine TCRαβ constant domains to increase the expression and pairing of the introduced TCRαβ chains.^32^^,^^39^ Furthermore, the TCRα and β chains are connected with a porcine-teschovirus P2A sequence. TCRαβ and CDR3αβ sequences of the HLA-E restricted TCRs recognizing the human cytomegalovirus UL40-derived epitope VMAPRTLIL were derived from the published HLA-E T cell clone KK50.4. *Mtb* or other CMV HLA-E restricted TCRαβ and CDR3αβ sequences were derived from HLA-E TM sorted T-cells and selected using the GLIPH2 cluster algorithm.^30^ TCR vector constructs were designed *in silico* with the software program Geneious Prime (version 2021.0). Synthesis and cloning of TCR constructs into the MP71flex retroviral vector were performed at BaseClear (Leiden, The Netherlands).

#### Transfections of Phoenix GALV packing cells

Human 293-based Phoenix GALV packaging cells were transfected with 2.6 μg MP71flex retroviral vector containing TCRαβ and CDR3αβ sequences recognizing previously identified *Mtb*- or CMV-derived peptides.^46^ Retroviral vectors were packaged using Fugene HD transfection reagent (Promega) in serum-free Opti-MEM I medium (Invitrogen). Virus supernatant was harvested 24 or 48 h post transfection. For all T cell transductions, fresh or thawed virus supernatant was spun down for 20 min 2000G at 4°C on 30 μg/mL RetroNectin (TaKaRa Bio) coated and 2% Human Serum Albumin (HSA) blocked non-tissue culture treated plates (Greiner bio-one CellStar). Virus supernatant was removed from the wells before transduction.

#### Transduction into J76 TPR CD8^+^/JE6.1 TPR TCR^ko^ CD8^+^ T cell lines

CD8^+^ TCR^ko^ Jurkat E6.1 or CD8^+^ Jurkat J76 triple parameter reporter (TPR) cell lines expressing three independent fluorescent reporter constructs which measure the activity of the transcription factors NFAT, NF-kB and AP-1, respectively^32^^,^^47^ were transduced with Phoenix-GALV virus supernatant for 24 h in 30 μg/mL RetroNectin coated and 2% HSA blocked non-tissue culture treated plates. Transduced CD8^+^ Jurkat cells were subsequently transferred to culture plates (Greiner Bio) and expanded. TCR expression was monitored 3 days post transduction using hamster anti-mouse constant TCRβ (APC-Cy7) (clone: H57-597 BioLegend) and mouse anti-human CD3 (BV510) (clone: UCHT1 BioLegend) monoclonal antibodies (moAbs) on a BD LSR Fortessa.

#### Transduction into isolated primary CD3^+^ T-cells

PBMCs were isolated from buffy coats following Ficoll-density gradient centrifugation. CD3^+^ T-cells were isolated via labeling with CD3 MicroBeads (Miltenyi) in an autoMACS Separator (Miltenyi) according to the manufacturer’s protocol. 3x10^5^ isolated CD3^+^ T-cells were cultured for 2 days together with 10^6^ irradiated autologous feeders in IMDM supplemented with 10% FCS, 25 Cetus units/mL IL-2 (Proleukin) and 2 μg/mL phytohemagglutinin (PHA) (Remel Europe Ltd.) at 37°C, 5% CO_2_. Expanded CD3^+^ T-cells were transduced overnight (O/N) in 30 μg/mL RetroNectin coated and 2% HSA blocked non-tissue culture treated plates and transferred to tissue-culture plates. Transduction efficiency was checked 48 h after transduction and transduced primary T-cells were used for stimulation experiments.

#### Zap70 activation of TCR-transduced Jurkat or primary T-cells

TCR-transduced Jurkat cells were either stimulated with K562 HLA-E^∗^01:03 presenting cells or with HLA-E^∗^01:03 tetramers. Transduced primary CD3^+^ T-cells were stimulated with peptide-loaded K562 cells. TCR induced activation was determined by Zap70 phosphorylation of tyrosines (Y) at positions 292 and 319. K562 HLA-E∗01:03 cells were peptide-loaded at 23°C O/N in a bead bath. 300.000 TCR transduced Jurkat cells or primary CD3^+^ T-cells were stained before stimulation with hamster anti-mouse constant TCRβ (APC-Cy7) (clone: H57-597 Biolegend), including CD3 (BV510) (clone: UCHT1, Biolegend), CD4 (BV650) (clone: OKT4 Biolegend) and CD8 (FITC) (clone: HIT8a, Biolegend) for primary CD3^+^ T-cells, for 30 min in PBS/0.1% BSA at 4°C in the dark. Jurkat/primary CD3^+^ T-cells were washed and stimulated with 50.000 K562 HLA-E^∗^01:03 cells loaded with 20 μg/mL peptide or 0.8 μg HLA-E∗01:03 tetramers (in the case of Jurkat cells) for 5 min at 37°C. After stimulation, 50 μL pre-warmed BD Cytofix Fixation Buffer (BD Bioscience) was added for 10 min to stop stimulation. Cells were then washed in Stain Buffer (BD Bioscience) and permeabilized in ice-cold BD Phosflow Perm Buffer III (BD Bioscience) for 30 min on ice. Cells were then washed twice in Stain Buffer and stained with anti-human PE-Cy7 Zap70 (pY319)/Syk (Y352) (clone: 17A/P-ZAP70 BD Bioscience) and anti-human PE Zap70 (pY292) (clone: J34-602 BD Bioscience) for 1 h at RT in the dark. Cells were fixated in 0.1% paraformaldehyde (PFA) and acquired on a BD LSR Fortessa (BD Bioscience). Analysis was done using FlowJo v10.8.1 software. Percentage peptide specific phosphorylation for transduced Jurkat and primary CD8^+^ T-cells -as determined via gating on the CD8^+^ T cell population within the transduced CD3^+^ T cell population-was calculated based on the mean fluorescent intensity (MFI) of phosphorylated Y292 and Y319 using the following formula: ((MFI_Y292/Y319_ TCR_p34orp55_ - MFI_Y292/Y319_ negative controls/(MFI_Y292/Y319_ negative controls)) ∗ 100%. Negative control K562-HLA-E stimulation: K562 cells loaded with control peptide p44. Negative control HLA-E TM stimulation: HLA-E TM loaded with p44. Representative gating strategies for Jurkat and primary T-cells are shown in Figure S4.

### Quantification and statistical analysis

Statistical details of experiments can be found in the figure legends. Data was tested for normality using the Anderson-Darlin test. Non-normal data was analyzed with the Mann-Whitney U test or non-parametric ANOVA test, depending on the number of groups compared. Normal data was analyzed with the one-way ANOVA test. Value of n represents the number of donors. Data is shown either as individual data points or the median value +/− 95% confidence interval.

## Supplemental information

Document S1. Figures S1–S5 and Table S1

Table S2. Complete data of TCR Vα and Vβ sequences and CDR3αβ sequences from all individuals from the RNAseq dataset, as further explained in the STAR Methods
