# Supplementary material for: Mtb HLA-E-tetramer-sorted CD8+ T cells have a diverse TCR repertoire
Source: iScience. 2024 Feb 15;27(3):109233. doi: 10.1016/j.isci.2024.109233 (PMC10909886; doi:10.1016/j.isci.2024.109233)
Supplement: Document S1. Figures S1–S5 and Table S1 [file mmc1.pdf]

iScience, Volume *27*

Supplemental information

Mtb HLA-E-tetramer-sorted CD8$^+$ T cells have a diverse TCR repertoire

Linda Voogd, Anne M.H.F. Drittij, Calinda K.E. Dingenouts, Kees L.M.C. Franken, Vincent van Unen, Krista E. van Meijgaarden, Paula Ruibal, Renate S. Hagedoorn, Judith A. Leitner, Peter Steinberger, Mirjam H.M. Heemskerk, Mark M. Davis, Thomas J. Scriba, Tom H.M. Ottenhoff, and Simone A. Joosten

SUPPLEMENTARY INFORMATION

SUPPLEMENTARY FIGURES AND LEGENDS

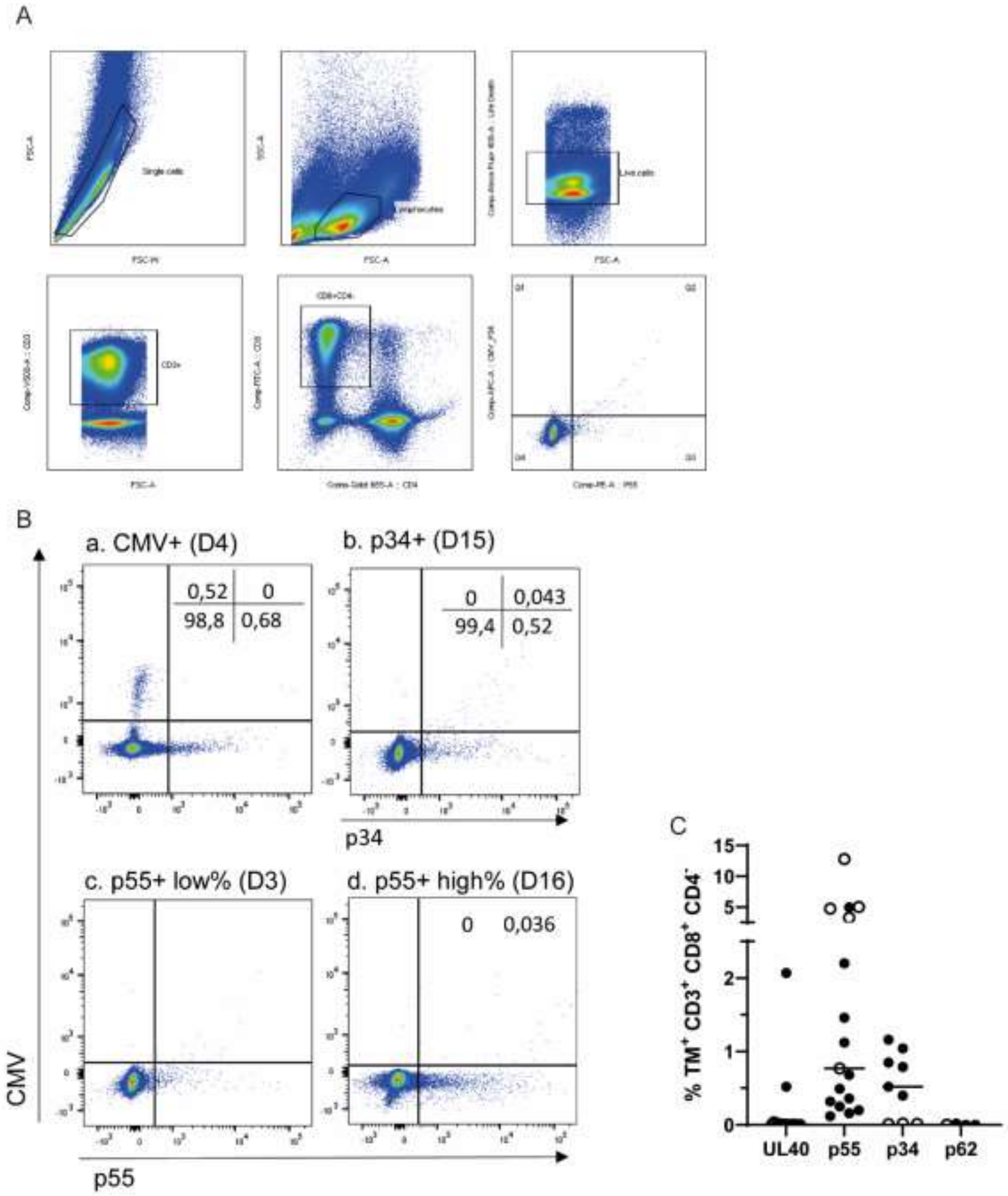

Figure S1. Gating strategy to sort HLA-E TM$^+$ CD8$^+$ T-cells, either for single cell sorting or for bulk sorting, related to Figure 1. (A) Gating strategy for sorting peptide specific populations: 1) single cells, 2) lymphocytes, 3) Live cells, 4) CD3$^+$ cells, 5) CD8$^+$CD4$^-$, 6) HLA-E TM specific population. **(B)** Representative dot plots for *ex vivo* TM staining for p62, p55, p34 or UL40 HLA-E CD8$^+$ T-cells (indicated on the y or x-axis). Shown are a. UL40$^+$ donor (D4), b. p34$^+$ donor (D16), c. p55^{low} donor (D15) and d. p55^{high} donor (D3). Percentages of cells are indicated per quadrant. **(C)** Combined mean percentage of peptide specific HLA-E CD8$^+$ T-cell population. Each dot represents one donor; samples sizes are between 50.000 and 350.000 CD3$^+$CD8$^+$CD4$^-$ live cells, except for open dots, indicating samples with 10-50.000 cells.

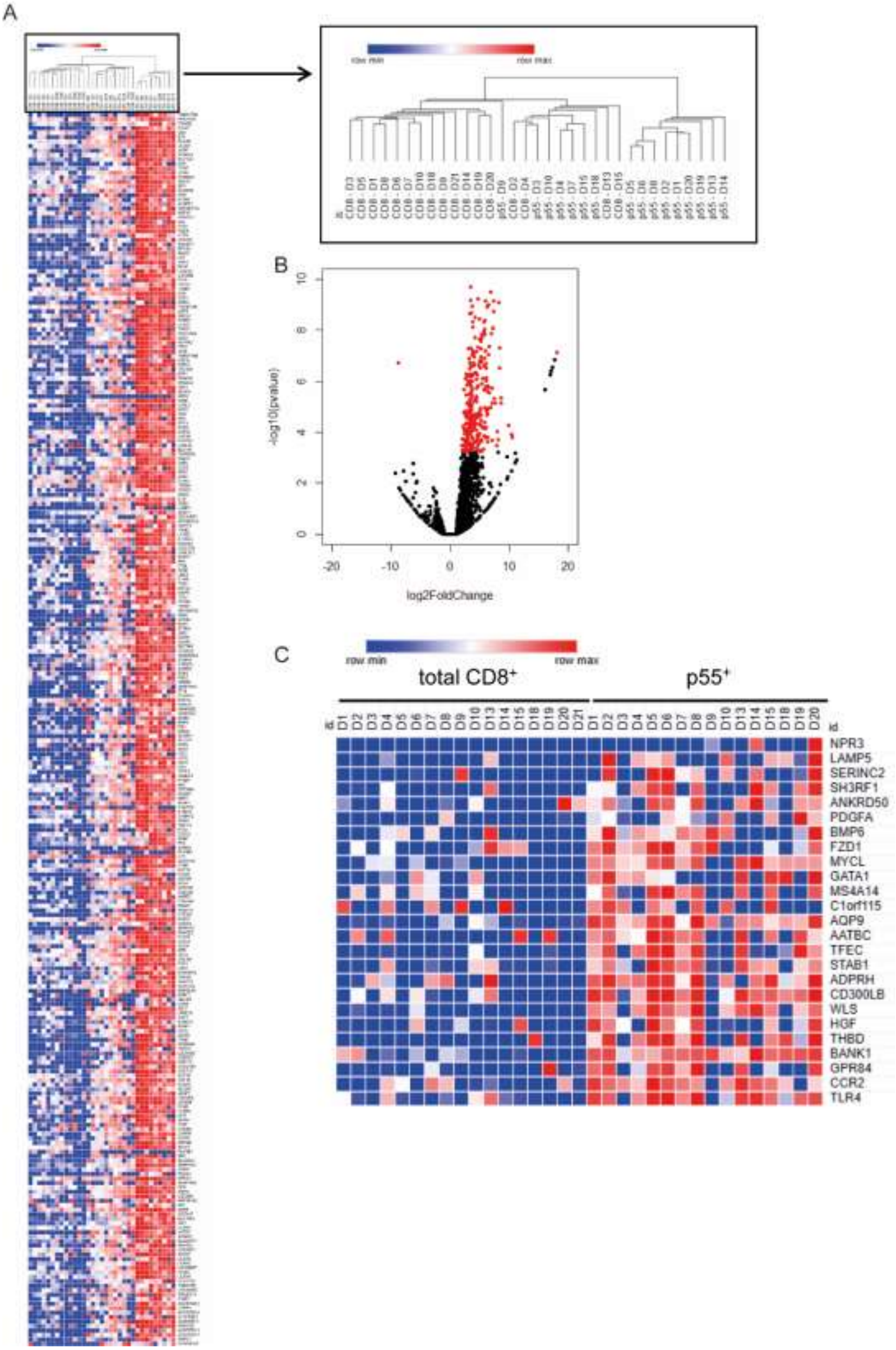

Figure S2. ***Mtb*** **p55 TM sorted T-cells have distinct transcriptomic profile compared to the bulk CD8^{+} TM^{-} T-cell population, derived from the RNA-seq dataset as explained in the STAR Methods.** PBMCs from South African adolescents, stained with HLA-E TM containing *Mtb* p55 (TMp55) were separated by flow cytometric sorting into CD8^{+} T-cells negative for TMp55 and CD8^{+} T-cells positive for TMp55. Sorted populations were analyzed by RNA-seq and differentially expressed genes were determined by DESeq2 analysis. **(A)** 276 genes were differentially expressed between

bulk CD8$^+$ TM$^-$ T-cells and T-cells stained with *Mtb* p55 loaded HLA-E TM, with parameters set at: adjusted p-value=0.05, LFC=1, counts>4 reads per gene. Data of the 276 differentially expressed genes were log2 transformed and clustered for similarities in gene expression patterns using clusterProfiler. **(B)** Volcano plot of the differentially expressed genes in one donor. Of the 276 genes with significant differential expression, 275 genes were upregulated, and 1 gene was downregulated. Log10 transformed P-values (Y-axis) were plotted versus the log2FoldChange (x-axis), dots in red represent genes with significant adjusted p-values (p-adj<0.05). **(C)** The top 25 genes, selected on the highest log-fold-change in differential expression shown in a heatmap for the CD8$^+$ TM$^-$ and CD8$^+$TMp55$^+$ populations from 16 individual donors. Read counts were log2 transformed and plotted with individual scaling per gene.

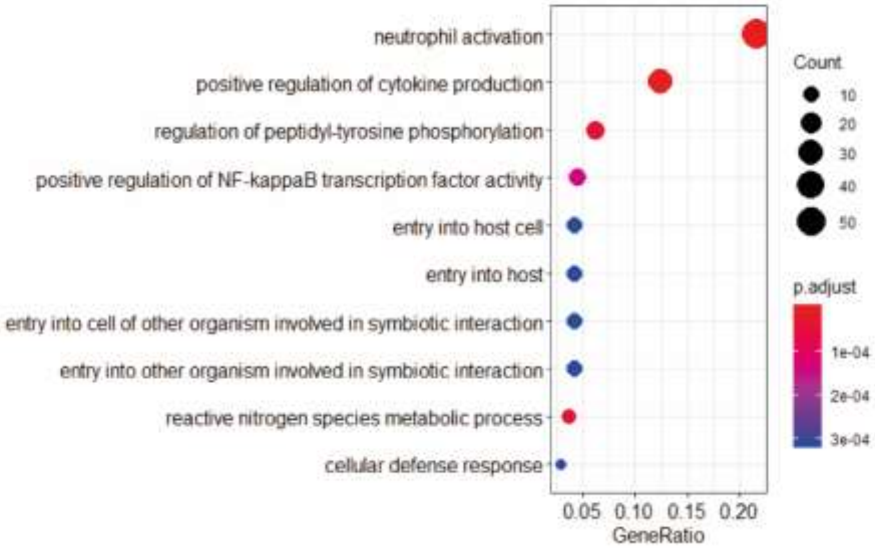

Figure S3. Gene Ontology enrichment analysis on bulk RNA-seq data of p55$^+$ HLA-E TM$^+$ T-cells and bulk CD8$^+$ TM$^-$ T-cells, derived from the RNA-seq dataset as explained in the STAR Methods.

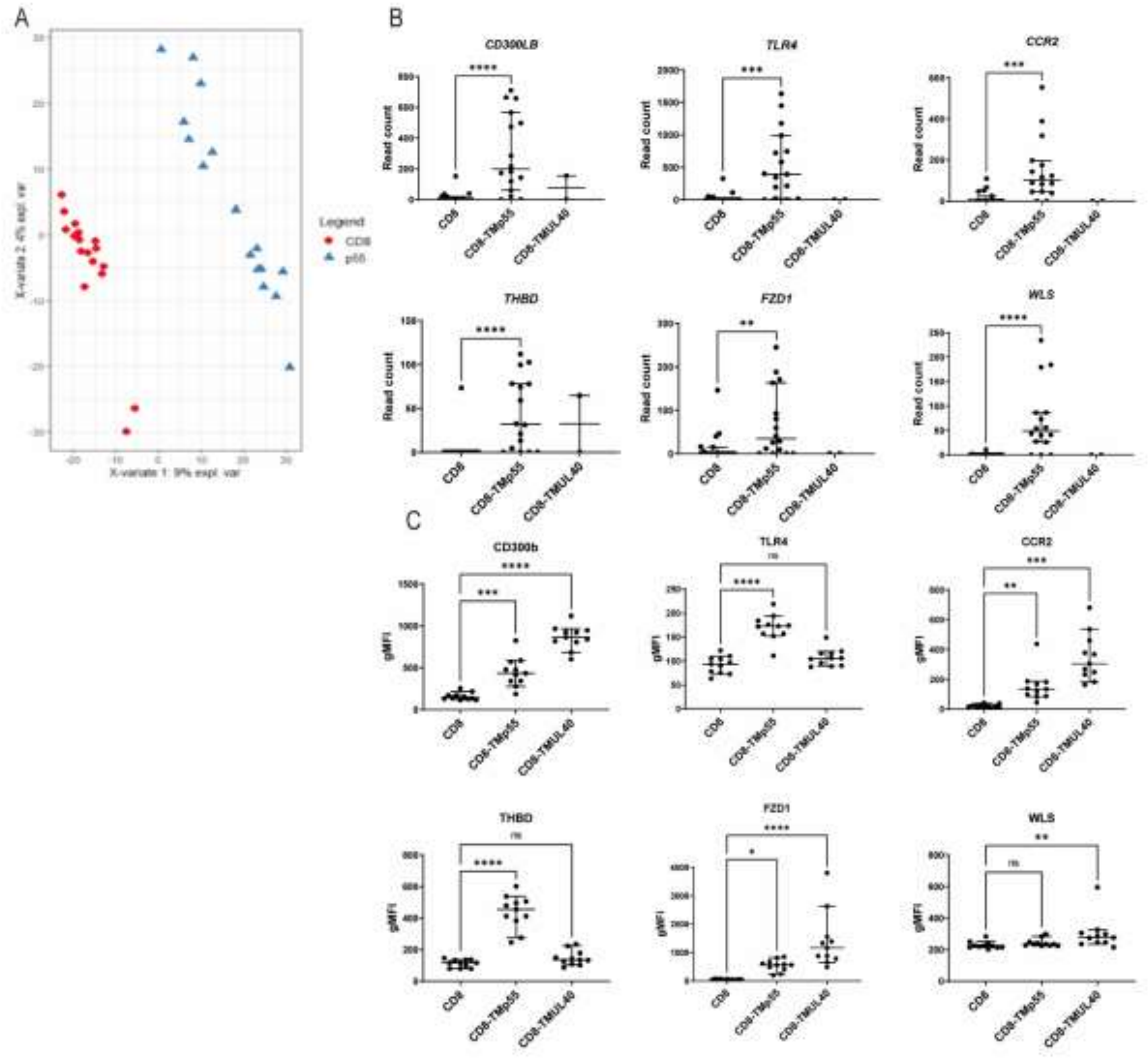

Figure S4. Differentially expressed gene analysis on bulk CD8$^+$ TM$^-$ T-cells, *Mtb* or UL40 HLA-E TM$^+$ T-cells, derived from the RNA-seq dataset as explained in the STAR Methods. (A) PCA discriminates the CD8$^+$ TM$^-$ (red circles) and CD8$^+$TMp55$^+$ (blue triangles) T-cell populations. **(B)** Median with 95% CI read counts from RNA sequencing data for the six genes shown for the total CD8$^+$ TM$^-$ T-cell population (n=16), TMp55 sorted T-cells (n=16) and TMUL40 sorted T-cells (n=2). Normality of 6 genes for CD8$^+$ TM$^-$ and TMp55 T-cells was tested with Anderson-Darlin revealing a non-normal distribution. Significance was calculated using Mann-Whitney U testing (p<0.05). Read count from TM$^+$ UL40 sorted T-cells were not included in the analysis because of the limited number of donors. **(C)** PBMCs from 11 independent South-African individuals were stained with HLA-E TMs loaded with p55 or UL40 in combination with moAbs against the selected markers depicted on the Y-axis to evaluate cell surface expression of the selected markers. Graphs show median MFI with 95% CI. Normality was tested with Anderson-Darling testing revealing non-normal distribution for WLS and FZD1. Significance was tested with one-way ANOVA, corrected for multiple comparisons (Dunnett) for normal distributed data and with non-parametric ANOVA (Friedman test) corrected for multiple comparisons (Dunnett) for non-normal distributed data (p<0.05).

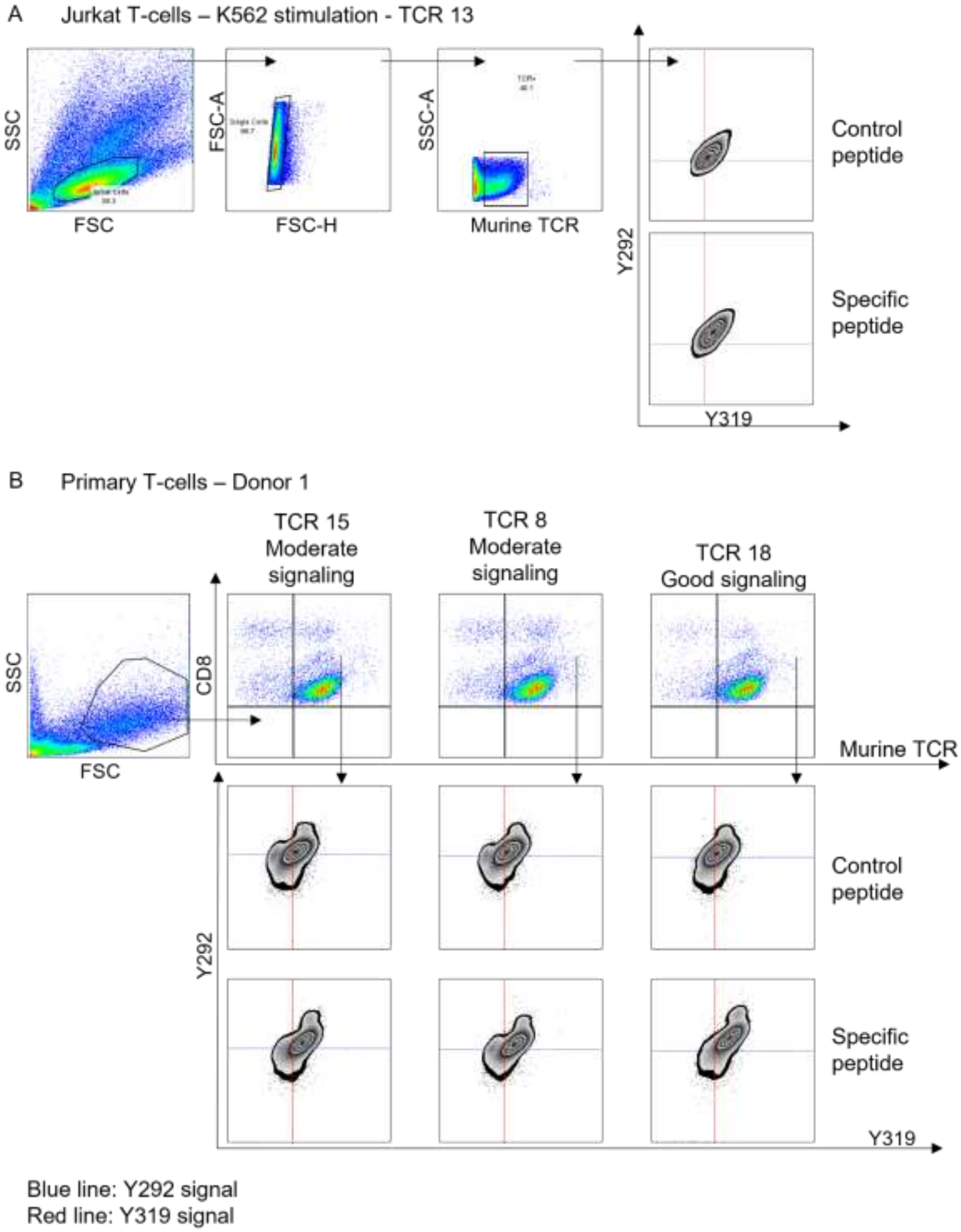

Figure S5. Gating strategy to determine Zap70 phosphorylation at Y292 and Y319 in both Jurkat cells and primary CD8⁺ T-cells, related to Figure 5. (A) Representative gating strategy for 1 TCR sorted for p34 (TCR 13) transduced in Jurkat cells after stimulation with peptide-loaded K562 cells: Jurkat cells, single cells, murine TCR^+ population (=transduced Jurkat cells) and $Y292^+$ and $Y319^+$ cell population according to the signal in the negative control condition. **(B)** Gating strategy for 3 TCRs with different percentage peptide specific phosphorylation transduced in primary $CD8^+$ T-cells from 1 donor after stimulation with peptide-loaded K562 cells: Jurkat cells attached to K562 cells, $CD8^+$ murine TCR^+ population and $Y292^+$ and $Y319^+$ cell population according to the signal in the negative control. condition.

SUPPLEMENTARY TABLES AND LEGENDS

Table S1. Details of recruited participants and sorted HLA-E TM$^+$ populations per participant as further outlined in the STAR methods.

Donor	Sex	Age	Ethnicity	QFT	QFT value	HLA-E genotype	TM combination 1	TM combination 2	Single cell sort
D1	f	16	black	pos	23,92	HLA-E*0101-0103	p55 vs UL40 (VMAPRTLLL)	p55 vs p62	p55
D2	f	14	black	neg	0,08	HLA-E*0101	p55 vs UL40 (VMAPRTLLL)	p55 vs p62	p55
D3	f	18	black	pos	10,03	HLA-E*0101-0103	p55 vs UL40 (VMAPRTLLL)	p55 vs p62	p55 and p62
D4	f	16	black	neg	0,16	HLA-E*0101	p55 vs UL40 (VMAPRTLLL)	p55 vs p62	p55 and p62
D5	f	16	black	pos	0,7	HLA-E*0101	p55 vs UL40 (VMAPRTLLL)	p55 vs p62	p55
D6	f	15	black	neg	0	HLA-E*0101-0103	p55 vs UL40 (VMAPRTLLL)	p55 vs p62	p55
D7	m	16	black	pos	35,45	HLA-E*0101	p55 vs UL40 (VMAPRTLLL)	p55 vs p62	p55
D8	f	16	black	neg	-0,01	HLA-E*0101	p55 vs UL40 (VMAPRTLLL)	p55 vs p62	p55
D9	m	16	black	neg	0,12	HLA-E*0101-0103	p55 vs UL40 (VLAPRTLLL)	p55 vs p34	p55 and p34
D10	m	18	black	pos	0,84	HLA-E*0101	p55 vs UL40 (VLAPRTLLL)	p55 vs p34	p55 and p34
D11	m	18	black	pos	0,59	HLA-E*0101-0103	p55 vs UL40 (VLAPRTLLL)	p55 vs p34	p55 and p34
D12	m	14	black	pos	15,1	HLA-E*0101	p55 vs UL40 (VLAPRTLLL)	p55 vs p34	p55 and p34
D13	f	15	black	neg	0	HLA-E*0101-0103	p55 vs UL40 (VLAPRTLLL)	p55 vs p34	p55 and p34
D14	m	16	black	neg	0,01	HLA-E*0101	p55 vs UL40 (VLAPRTLLL)	p34 vs UL40 (VLAPRTLLL)	p55 and p34
D15	m	14	black	neg	-0,09	HLA-E*0101	p55 vs UL40 (VLAPRTLLL)	p34 vs UL40 (VLAPRTLLL)	p55 and p34
D16	m	16	black	pos	12,02	HLA-E*0101	p55 vs UL40 (VLAPRTLLL)	p34 vs UL40 (VLAPRTLLL)	p55 and p34
D17	f	14	black	pos	1,46	HLA-E*0101	p55 vs UL40 (VLAPRTLLL)	p34 vs UL40 (VLAPRTLLL)	p55 and p34
D18	m	15	black	pos	2,96	HLA-E*0101-0103	p55 vs UL40 (VLAPRTLLL)	p34 vs UL40 (VLAPRTLLL)	p55 and p34
D19	m	18	black	pos	74,86	HLA-E*0101-0103	p55 vs UL40 (VLAPRTLLL)	p34 vs UL40 (VLAPRTLLL)	p55 and p34
D20	f	14	black	neg	0	HLA-E*0101-0103	p55 vs UL40 (VLAPRTLLL)	p34 vs UL40 (VLAPRTLLL)	p55 and p34
D21	f	16	black	neg	-0,07	HLA-E*0101	p55 vs UL40 (VLAPRTLLL)	p34 vs UL40 (VLAPRTLLL)	p55 and p34
